# Political optimizer with interpolation strategy for global optimization

**DOI:** 10.1371/journal.pone.0251204

**Published:** 2021-05-06

**Authors:** Aijun Zhu, Zhanqi Gu, Cong Hu, Junhao Niu, Chuanpei Xu, Zhi Li

**Affiliations:** 1 School of Electronic Engineering and Automation, Guilin University of Electronic Technology, Guilin, P.R. China; 2 Department of Computer Science and Engineering, Texas A&M University, College Station, TX, United States of America; 3 School of Mechano-Electronic Engineering, Xidian University, Xi’an, P.R. China; 4 Guangxi Key Laboratory of Automatic Detecting Technology and Instruments, Guilin, P.R. China; 5 Guilin University of Aerospace Technology, Guilin, P.R. China; University of Lincoln, UNITED KINGDOM

## Abstract

Political optimizer (PO) is a relatively state-of-the-art meta-heuristic optimization technique for global optimization problems, as well as real-world engineering optimization, which mimics the multi-staged process of politics in human society. However, due to a greedy strategy during the election phase, and an inappropriate balance of global exploration and local exploitation during the party switching stage, it suffers from stagnation in local optima with a low convergence accuracy. To overcome such drawbacks, a sequence of novel PO variants were proposed by integrating PO with Quadratic Interpolation, Advance Quadratic Interpolation, Cubic Interpolation, Lagrange Interpolation, Newton Interpolation, and Refraction Learning (RL). The main contributions of this work are listed as follows. (1) The interpolation strategy was adopted to help the current global optima jump out of local optima. (2) Specifically, RL was integrated into PO to improve the diversity of the population. (3) To improve the ability of balancing exploration and exploitation during the party switching stage, a logistic model was proposed to maintain a good balance. To the best of our knowledge, PO combined with the interpolation strategy and RL was proposed here for the first time. The performance of the best PO variant was evaluated by 19 widely used benchmark functions and 30 test functions from the IEEE CEC 2014. Experimental results revealed the superior performance of the proposed algorithm in terms of exploration capacity.

## Introduction

Global optimization problems (GOPs) are inevitable in applied mathematics and practical engineering fields. As a general rule, most GOPs can be formulated as follows:

$$\min\;f\left(x\right),x=\left(x_{1},x_{2},\ldots ,x_{n}\right)$$ (1)

where *f*(*x*) and *n* denote an objective function and the number of variables, respectively. *R* is the real field, *x*∈*Q* and *Q* is an n-dimensional rectangle in *R*^*n*^ defined by the following equation:

$$Q=\Pi_{i=1}^{n}\left[l_{i},u_{i}\right]$$ (2)

Where *l* = (*l*_1_,…,*l*_*n*_), u = (*u*_1_,…,*u*_*n*_), *x*_*i*_∈(*l*_*i*_, *u*_*i*_), *i* = 1,…,*n* and [*l*, *u*] is the feasible region.

Over the last two to three decades, meta-heuristic optimization techniques have been extremely popular in the GOPs field. Generally speaking, meta-heuristic optimization techniques may be divided into four categories: swarm-based, evolution-based, human behavior-based, and physics-based algorithms [1, 2]. A few well-known and state-of-the-art swarm-based algorithms primarily include Salp Swarm Optimizer [3], Grey Wolf Optimizer (GWO) [4], Particle Swarm Optimization (PSO) [5], Ant Colony Optimization [6], and [7–24], etc. Well-known evolution-based algorithms mainly include biogeography based optimizer [25], Differential Evolution (DE) [26], Evolutionary Strategy [27], Genetic Programming [28], and Genetic Algorithm [29]. Known human behavior-based algorithms primarily include Brain Strom Optimization [30], Soccer League Competition [31], and [32–38], etc. Familiar physics-based algorithms include Thermal Exchange Optimization [39], Gravitational Search Algorithm [40], Sine Cosine Algorithm (SCA) [41], and [42–50], etc.

Although Sorensen presented a more critical view on such meta-heuristic methods in 2015 [51], this did not prevent researchers from steadily creating new methods based on meta-heuristics. Since No Free Lunch Theory [52] gives the following enlightenment: there are no algorithms which can solve all problems, this motivates researchers develop new meta-heuristic algorithms ceaselessly. Recently (2020), Askari proposed a new meta-heuristic method called political optimizer (PO), which was inspired by a multi-staged process of politics and described a model to solve GOPs [53]. PO is the mathematical mapping of all the major phases of politics [54–56]; thus, it belongs to human behavior-based algorithms. Experimental results have demonstrated that PO can solve classical engineering design problems, such as welded beam and speed reducer design. Results have indicated that the PO had excellent convergence speed performance, and a good exploration capacity in early iterations.

However, almost all individuals in the canonical PO are concentrated in a narrow area near the current optimal individual in the final stage of iterative optimization. Therefore, when solving complex multimodal global optimization problems, the entire population can easily converge to the local optimum. How to improve the capacity for jumping out of the local optimum in PO is the key technology, and the most important goal of global optimization. There are two main methods for improving the ability to jump out of the local optimum in PO. The first involves adjusting the parameters in PO, whereas the second is to introduce new search operators. However, there is only one parameter n (number of political parties, constituencies, and party members). According to the canonical PO, n should be set at 8 to obtain an appropriate convergence; thus, it is the better choice when introducing new search operators.

Furthermore, canonical PO has an inappropriate balance of global exploration and local exploitation during the party switching stage. In the canonical PO, the values of λ are linearly decreased from one to zero; however, it is not appropriate to adopt a linear parameter strategy to simulate the actual nonlinear search process.

Consequently, a summative conclusion may be drawn as follows. Firstly, the limited global optima searching capacity of canonical PO makes it stagnate in a local optimum with high probability and may lead to premature convergence. Second, it is not appropriate to adopt a linear parameter strategy to simulate the nonlinear search process, which leads to an unsuitable balance of global exploration and local exploitation during the party switching stage. Finally, due to limited iterations, the global optima in canonical PO are difficult to find as relates to complicated multimodal problems. These conclusions motivated us to make the following innovative implementation, with the main contributions of this work listed as follows:

1. An interpolation strategy was adopted to facilitate the current global optima jump out of local optima. Interpolation is the process of synthesizing all known data to predict unknowns, which can take full advantage of certain known data.
2. If interpolation strategy is utilized solely, the diversity of the population quickly declines. Therefore, a refraction learning (RL) strategy was adopted to improve the population diversity.
3. To enhance the capacity of balancing exploration and exploitation during the party switching stage, a logistic model was proposed to maintain good balance.
4. To the best of our knowledge, PO combined with interpolation strategy and RL was proposed here for the first time.

This paper is further organized as below. Section 2 introduces preliminary knowledge of canonical PO. The proposed methodology is introduced in Section 3. In Section 4, 19 well-known and extensively employed benchmark functions and 30 test functions from the IEEE CEC 2014 were utilized to evaluate the proposed algorithms. Finally, Section 4 also summarizes with concluding remarks.

## Political optimizer (PO)

**Fig 1:**
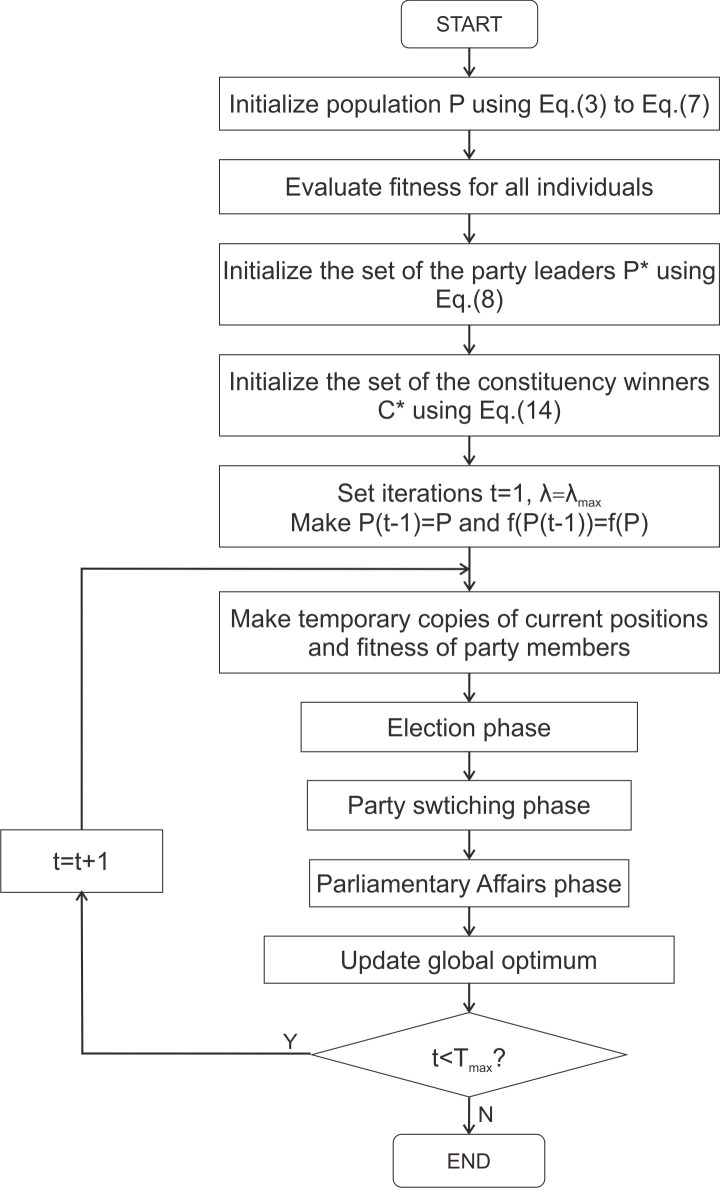
Main step of original PO.

**Fig 2:**
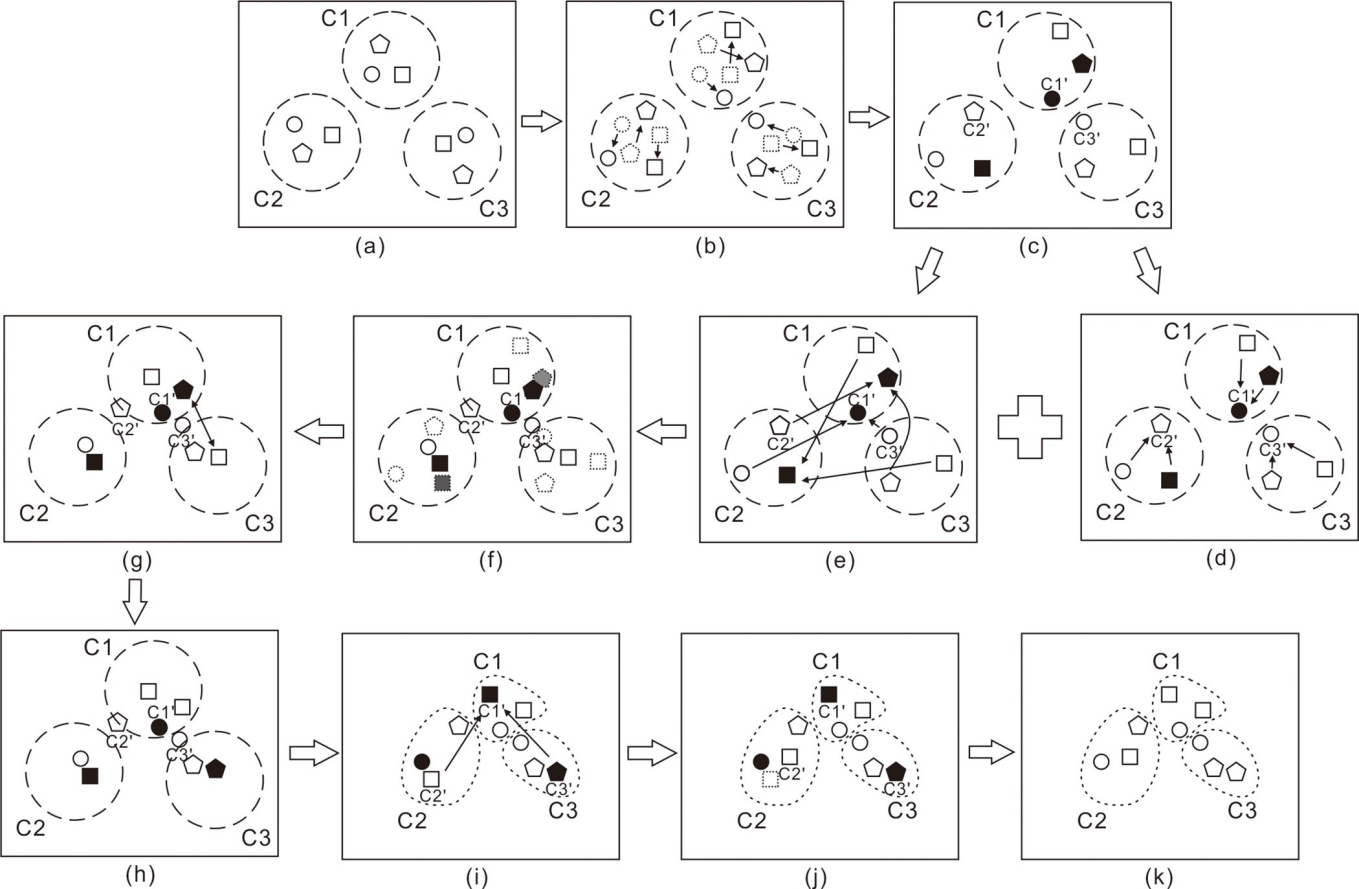
PO process. (A) Population initialization, different shapes refer to different parties. Quadrilaterals, pentagons, and circles represent political parties P_1_, P_2_, and P_3_, respectively. Dotted frames C1, C2, and C3 represent different constituencies. (B) Members of various political parties conduct canvassing activities within their respective constituency. (C) Party leaders (solid) and constituency winners C1’, C2’, and C3’ are determined. (D) Update the positions of party members according to the constituency winners. (E) Update the positions of party members according to the party leaders. (F) The resultant positions are synthesized according to the position of the constituency winners and the party leaders. (G)(H) party switch, $p_{1}^{3}$ in constituency C3 is exchanged with $p_{2}^{1}$ in constituency C1. (I) election phrase and reassign party leaders and constituency winners. (J) parliamentary affairs: each parliamentarian is updated if its fitness is improved after being attracted by a random parliamentarian. (K) final positions after one iteration.

PO is inspired by the western political process of optimization, which involves two aspects. The first assumption is that all citizens attempt to optimize their goodwill to win the election. The second assumption is that all parties try to obtain more seats in parliament. PO is consisted of five phases, which include party formation and constituency allocation, election campaign, party switching, interparty election, and parliamentary affairs [53]. The main PO process is shown in Fig 1, whereas the process of the system to express how the system works is shown in Fig 2.

The entire population can be divided into *n* political parties, which can be represented as Eq (3).

$$P=\left\{P_{1},P_{2},P_{3},\ldots ,P_{n}\right\}$$ (3)

Every party consists of *n* party members, as demonstrated in Eq (4).

$$P_{i}=\left\{p_{i}^{1},p_{i}^{2},p_{i}^{3},\ldots ,p_{i}^{n}\right\}$$ (4)

Each party member consists of *d* dimensions, as shown in Eq (5).

$$p_{i}^{j}={\left[p_{i,1}^{j},p_{i,2}^{j},p_{i,3}^{j},\ldots ,p_{i,d}^{j}\right]}^{T}$$ (5)

Each solution can also be an election candidate. Suppose there are *n* electoral districts as represented in Eq (6).

$$C=\left\{C_{1},C_{2},C_{3},\ldots ,C_{n}\right\}$$ (6)

It is assumed there are *n* members in each constituency, as shown in Eq (7).

$$C_{j}=\left\{p_{1}^{j},p_{2}^{j},p_{3}^{j},\ldots ,p_{n}^{j}\right\}$$ (7)

The party leader is defined as the member with the best fitness in a party, as shown in Eq (8).

$$\begin{array}{l} q=\underset{1\leq j\leq n}{argmin}\;f\left(p_{i}^{j}\right),\forall i\epsilon \left\{1,\ldots ,n\right\}\\ p_{i}^{*}=p_{i}^{q} \end{array}$$ (8)

All of the party leaders can be expressed as Eq (9).

$$P^{*}=\left\{p_{1}^{*},p_{2}^{*},p_{3}^{*},\ldots ,p_{n}^{*}\right\}$$ (9)

The winners of the different constituencies are called members of parliament, as shown in Eq (10).

$$C^{*}=\left\{c_{1}^{*},c_{2}^{*},c_{3}^{*},\ldots ,c_{n}^{*}\right\}$$ (10)

During the election campaign stage, Eq (11) and Eq (12) are employed to update the position of potential solutions.

$$p_{i,k}^{j}(t+1)=\{\begin{array}{c} \mathrm{if}\;p_{i,k}^{j}(t-1)\leq p_{i,k}^{j}(t)\leq m^{*}\;\mathrm{or}\;p_{i,k}^{j}(t-1)\geq p_{i,k}^{j}(t)\geq m^{*},\\ m^{*}+r\left(m^{*}-p_{i,k}^{j}(t)\right);\\ \mathrm{if}\;p_{i,k}^{j}(t-1)\leq m^{*}\leq p_{i,k}^{j}(t)\;\mathrm{or}\;p_{i,k}^{j}(t-1)\geq m^{*}\geq p_{i,k}^{j}(t),\\ m^{*}+(2r-1)\left|m^{*}-p_{i,k}^{j}(t)\right|;\\ \mathrm{if}\;m^{*}\leq p_{i,k}^{j}(t-1)\leq p_{i,k}^{j}(t)\;\mathrm{or}\;m^{*}\geq p_{i,k}^{j}(t-1)\geq p_{i,k}^{j}(t),\\ m^{*}+(2r-1)\left|m^{*}-p_{i,k}^{j}(t-1)\right|; \end{array}$$ (11)

$$p_{i,k}^{j}(t+1)=\{\begin{array}{c} \mathrm{if}\;p_{i,k}^{j}(t-1)\leq p_{i,k}^{j}(t)\leq m^{*}\;\mathrm{or}\;p_{i,k}^{j}(t-1)\geq p_{i,k}^{j}(t)\geq m^{*},\\ m^{*}+(2r-1)\left|m^{*}-p_{i,k}^{j}(t)\right|;\\ \mathrm{if}\;p_{i,k}^{j}(t-1)\leq m^{*}\leq p_{i,k}^{j}(t)\;\mathrm{or}\;p_{i,k}^{j}(t-1)\geq m^{*}\geq p_{i,k}^{j}(t),\\ p_{i,k}^{j}(t-1)+r\left(p_{i,k}^{j}(t)-p_{i,k}^{j}(t-1)\right);\\ \mathrm{if}\;m^{*}\leq p_{i,k}^{j}(t-1)\leq p_{i,k}^{j}(t)\;\mathrm{or}\;m^{*}\geq p_{i,k}^{j}(t-1)\geq p_{i,k}^{j}(t),\\ m^{*}+(2r-1)\left|m^{*}-p_{i,k}^{j}(t-1)\right|; \end{array}$$ (12)

To balance exploration and exploitation, party switching is adopted. An adaptive parameter λ is used, which is linearly decreased from one to zero during the entire iterative process. Each candidate is selected according to probability λ and exchanged with the worst member of a randomly selected party, as shown in Eq (13).

$$q=\underset{1\leq j\leq n}{argmax}\;f\left(p_{i}^{j}\right)$$ (13)

In the election phrase, the winner in a constituency is obtained, as shown in Eq (14).

$$\begin{array}{l} q=\underset{1\leq j\leq n}{argmin}\;f\left(p_{i}^{j}\right)\\ \;c_{j}^{*}=p_{q}^{j} \end{array}$$ (14)

## Proposed methodology

PO can obtain good convergence when dealing with simple problems; however, it easily falls into local optima when engaged with multi-peak test benchmark functions. To more efficiently obtain an optimum, interpolation strategy is introduced into the PO.

Interpolation strategy is based on known discrete points, where the interpolation method employs the data of known points to obtain other unknown points. It can obtain information on unknown points by employing the data of known points, so that it can make full use of the data of the known points to obtain an optimal solution in PO. The introduction of the interpolation can overcome the shortcomings of PO, which include its easily falling into local optima. In this section, several interpolation strategies were applied to PO, wherein the most suitable interpolation strategy was selected to combine with RL according to the results of various interpolation strategies.

**Fig 3:**
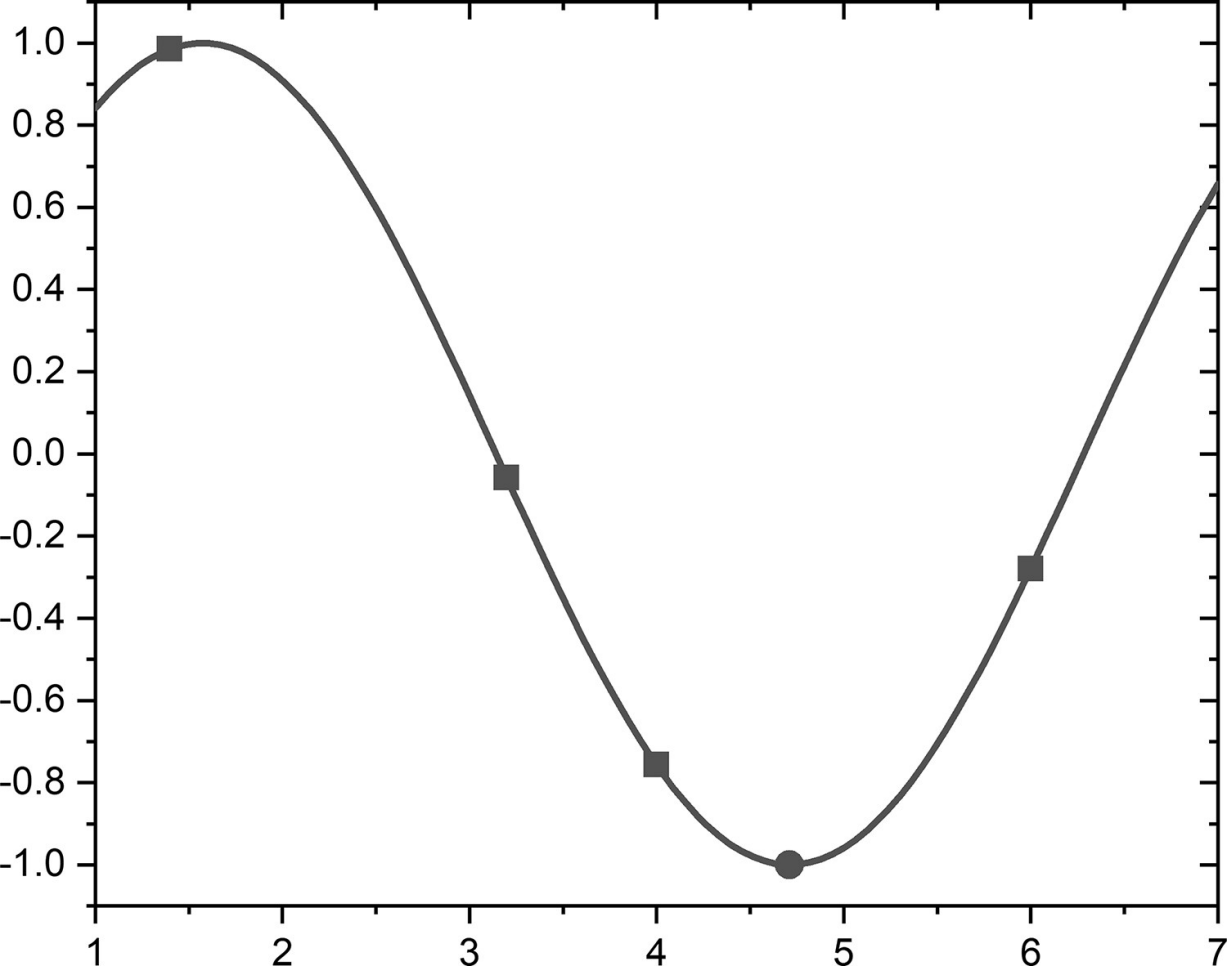
Example of interpolation.

The interpolation strategy is shown in Fig 3, where quadrilaterals represent known discrete points, solid lines are continuous functions drawn according to the data of known points, and circles represent the position where the optimal point is predicted (the lowest point in this example), where the lowest point is contingent on the constructor method. Following the use of the interpolation strategy a derived optimal solution is generated and compared with the solution generated by the original PO. If the solution is better than that generated by the PO, it will be replaced; otherwise, the solution generated by PO will be retained. This method was used for each of the interpolation strategies in this section and not be repeated.

### Quadratic interpolation PO (QIPO)

Quadratic interpolation is one of the most common strategies used in unconstrained one-dimensional optimization. In the case that the current optimal solution is known, the new optimal solution is explored based on other data. By using this approach, the known data may be employed to calculate the real optimal solution. Quadratic interpolation is a curve fitting technique used to construct a quadratic function via the data of known point. The optimal point, and any two points of the contemporary population are selected to construct the quadratic function, and the optimal point of the quadratic function is obtained by Eq (15).

$$Q_{ni}=\frac{1}{2}\left[\frac{\left(x_{2i}^{2}-x_{3i}^{2}\right)f_{1}+\left(x_{3i}^{2}-x_{1i}^{2}\right)f_{2}+\left(x_{1i}^{2}-x_{2i}^{2}\right)f_{3}}{\left(x_{2i}-x_{3i}\right)f_{1}+\left(x_{3i}-x_{1i}\right)f_{2}+\left(x_{1i}-x_{2i}\right)f_{3}}\right]$$ (15)

**Fig 4:**
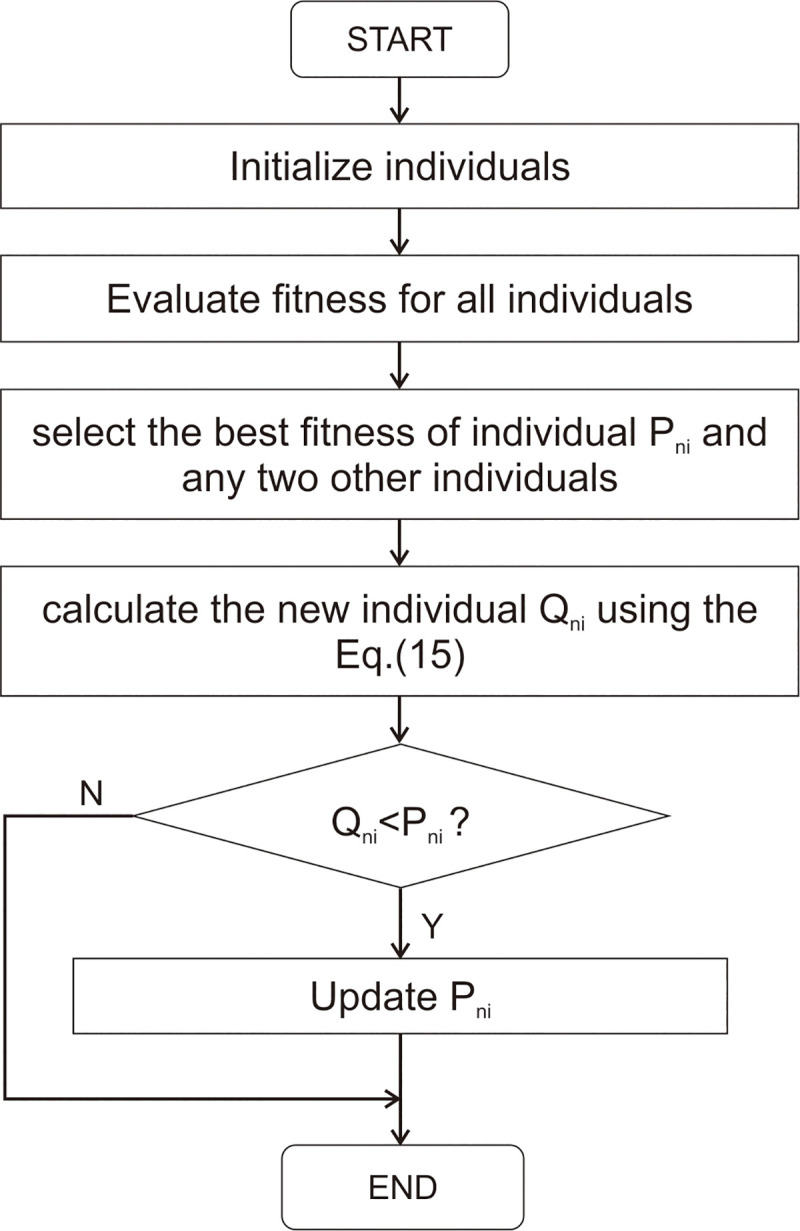
The QIPO process.

Where *x*_*1*_ *= (x*_*11*_,*x*_*12*_,*…*,*x*_*1i*_*)* represents the optimal individual of the contemporary population, *x*_*2*_ *= (x*_*21*_,*x*_*22*_,*…*,*x*_*2i*_*)* and *x*_*3*_ *= (x*_*31*_,*x*_*32*_,*…*,*x*_*3i*_*)* represents the other two individuals of the contemporary population; *f*_*1*,_ *f*_*2*_ and *f*_*3*_ are the fitness values of corresponding individual, respectively; *i* = 1,2, …,dim, dim is the maximum dimension. Quadratic interpolation is applied in each iteration, if the fitness value of optimal solution *f(Q*_*n*_*)* is better than that of the original optimal individual, the optimal individual is updated; otherwise, the original optimal individual is retained for the next iteration. The process of quadratic interpolation is shown in Fig 4.

Initially, PO initializes individuals in the first generation and calculates the fitness of all individuals. In the next step, it ranks the individuals by fitness and marks the best individual, and then randomly selects two individuals from all those except the optimal individual. These three individuals are selected to predict the best individual via interpolation strategy. Finally, the fitness value of the new individual is compared with the original optimal individual, after which the original individual is either retained or replaced according to the results of the comparison.

### Advance quadratic interpolation PO (AQIPO)

Similar to quadratic interpolation, advance quadratic interpolation utilizes other means to derive the object function. In such a strategy, the selection of any contemporary population transforms the constructed quadratic function into an arc function. This enhanced interpolation strategy further improves the accuracy of the results and does not increase the complexity of the algorithm. The object function *f(x)* is constructed by individuals *x*_**1**_, *x*_**2**_ and *x*_**3,**_ these individuals, *x*_**1**_ *= (x*_**11**_,*x*_**12**_,*…*,*x*_**1i**_*)*, *x*_**2**_ *= (x*_**21**_,*x*_**22**_,*…*,*x*_**2i**_*)*, *x*_**3**_ *= (x*_**31**_,*x*_**32**_,*…*,*x*_**3i**_*)*; *i* is the maximum dimension, whereas *f*_**1**_, *f*_**2**_ and *f*_**3**_ are the fitness values of corresponding individuals, respectively, Subsequently, we can obtain the arc equation F(x):

$$\left|\begin{array}{cccc} x^{2}+F^{2} & x & F & 1\\ x_{1}^{2}+f_{1}^{2} & x_{1i} & f_{1} & 1\\ x_{2i}^{2}+f_{2}^{2} & x_{2i} & f_{2} & 1\\ x_{3i}^{2}+f_{3}^{2} & x_{3i} & f_{3} & 1 \end{array}\right|=0$$ (16)

Expanding this matrix and taking its derivative with respect to *x*, gives *F’(x)* = 0, which can then obtain the optimal solution to the function:

$$Q_{ni}=\frac{1}{2}\left[\begin{array}{c} \frac{\left(x_{2i}^{2}-x_{3i}^{2}\right)f_{1}+\left(x_{3i}^{2}-x_{1i}^{2}\right)f_{2}+\left(x_{1i}^{2}-x_{2i}^{2}\right)f_{3}}{\left(x_{2i}-x_{3i}\right)f_{1}+\left(x_{3i}-x_{1i}\right)f_{2}+\left(x_{1i}-x_{2i}\right)f_{3}}\\ +\frac{\left(f_{1}-f_{2}\right)\left(f_{2}-f_{3}\right)\left(f_{3}-f_{1}\right)}{\left(x_{2i}-x_{3i}\right)f_{1}+\left(x_{3i}-x_{1i}\right)f_{2}+\left(x_{1i}-x_{2i}\right)f_{3}} \end{array}\right]$$ (17)

The fitness value of the optimal solution is compared with that of the optimal individual of the original population, where the best one is retained, and the position of the individual is updated. The process of advance quadratic interpolation is similar to quadratic interpolation, which only requires the replacement of Eq (15) with Eq (17) (Fig 4).

### Cubic interpolation PO (CIPO)

**Fig 5:**
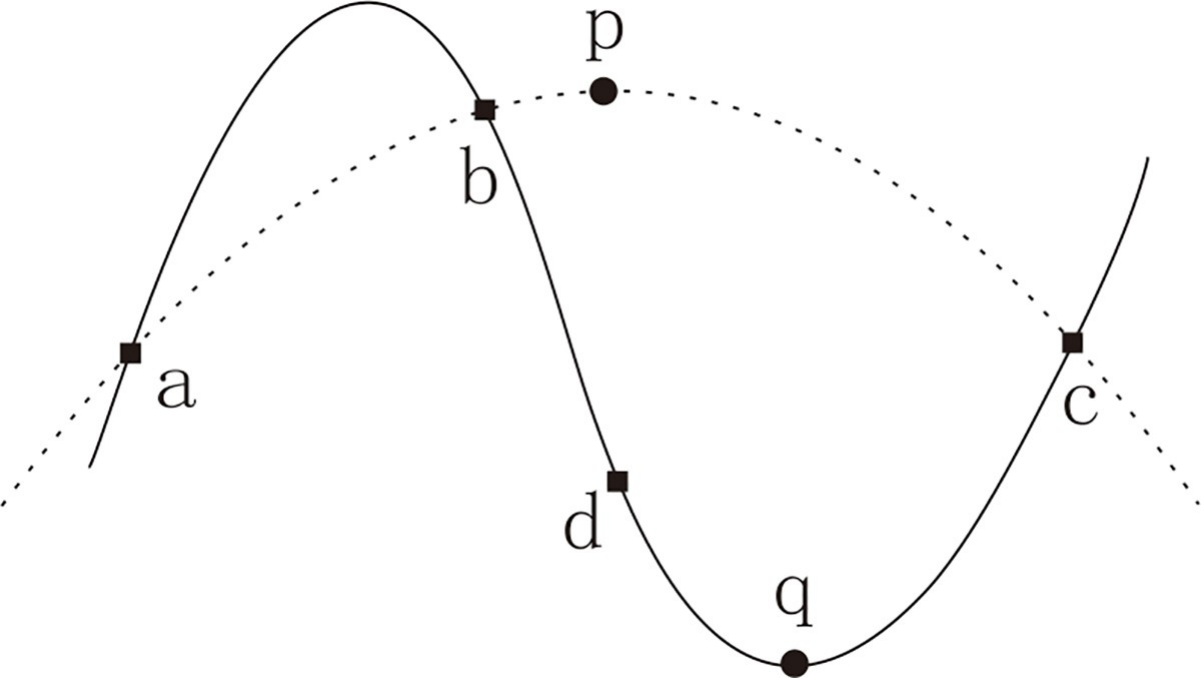
Cases when QI and AQI deal with multimodal problems.

There is an issue with quadratic interpolation and its improved strategies, as shown in Fig 5. The dashed line is the predict line of quadratic interpolation and solid line is the object function line. Point p is the optimal value inferred by the quadratic interpolation based on the information of points a, b, and c. There is quite a difference between point p and the object function point q, and this problem is unavoidable in quadratic interpolation.

To solve this problem, cubic Interpolation was adopted, which constructs a cubic function and obtains a new optimal solution by four known points, including the individual of the contemporary population. As the second derivative of the cubic function is introduced on the basis of Hermitian interpolation, the precision and stability of cubic interpolation are improved. Moreover, cubic interpolation utilizes additional data than quadratic interpolation so as to avoid the false optimum. The cubic interpolation method selects the optimal individuals *x*_*1*_ and three other individuals *x*_*2*_, *x*_*3*_ and *x*_*4*_ to represent all the information of the population. The four selected individuals are sequentially arranged (from small to large) to obtain the following formula:

$$h_{ki}=x_{\left(k+1\right)i}-x_{ki}$$ (18)

According to the continuity of interpolation:

$$L_{ki}\left(x_{ki}\right)=y_{ki}$$ (19)

$$L_{ki}\left(x_{\left(k+1\right)i}\right)=y_{\left(k+1\right)i}$$ (20)

Differential equation of cubic spline interpolation curve:

$$L_{ki}^{\prime }\left(x_{\left(k+1\right)i}\right)=L_{\left(k+1\right)i}^{\prime }\left(x_{\left(k+1\right)i}\right)$$ (21)

$$L_{ki}^{\prime \prime }\left(x_{\left(k+1\right)i}\right)=L_{\left(k+1\right)i}^{\prime \prime }\left(x_{\left(k+1\right)i}\right)$$ (22)

According to the differential equation of the cubic interpolation curve:

$$L_{ki}\left(x\right)=a_{ki}+b_{ki}\left(x-x_{ki}\right)+c_{ki}{\left(x-x_{ki}\right)}^{2}+d_{ki}{\left(x-x_{ki}\right)}^{3}$$ (23)

$$L_{ki}^{\prime }\left(x\right)=b_{ki}+2c_{ki}\left(x-x_{ki}\right)+3d_{ki}{\left(x-x_{ki}\right)}^{2}$$ (24)

$$L_{ki}^{\prime \prime }\left(x\right)=2c_{ki}+6d_{ki}\left(x-x_{ki}\right)$$ (25)

Combine Eq (18) to Eq (25), it can get a function for each interval by Eq (26):

$$Q_{ki}=3(\frac{h_{ki}}{h_{(k-1)i}+h_{ik}}\cdot \frac{y_{ki}-y_{(k-1)i}}{h_{(k-1)i}}+\frac{h_{(k-1)i}}{h(k-1)i+h_{ki}}\cdot \frac{y_{(k+1)i}-y_{ki}}{h_{ki}})$$ (26)

where *k* = 1, 2, 3; *x*_*1*_, *x*_*2*_, *x*_*3*_ and *x*_*4*_ optimal individuals of the contemporary population and any other three individuals, respectively. *y*_*1*_, *y*_*2*_, *y*_*3*_ and *y*_*4*_ are the fitness values of the corresponding individuals, respectively. *i* is the dimension from 1 to the maximum dimension. *Q*_*ki*_ represents the cubic function that corresponds to each subinterval. By connecting the functions of each subinterval, the continuous function that contains all the above points and the optimal solution *Q*_*ki*_ can be calculated. Finally, the fitness value *f(Q*_*k*_*)* of the new solution is compared with the fitness of the original optimal individual. If the fitness value of the new solution is better than that of the original optimal individual, the optimal individual is updated. PO with cubic interpolation requires more data than other interpolation strategies.

### Lagrange interpolation PO (LIPO)

To overcome the shortcomings of the original PO falling into local optima, PO with Lagrange interpolation was proposed. The principle involves the construction of quadratic functions from the data of known points, as well as other interpolations but is different from quadratic and cubic interpolation. Lagrange interpolation is not simply the solving of equations in that the conic formed by three points can be solved without constructing an equation. It can obtain the object function from the three quadratic curves via the following solution process:

Considering the unknown formula y_**ki**_ = ax^**2**^_**ki**_+bx_**ki**_+c, the quadratic function may be obtained by adding three quadratic functions that are derived from the data of the three known points. When *k =* 1, *y*_*1i*_ *=* 1, *y*_*2i*_ *= y*_*3i*_ *=* 0, the formula contains these points as *f*_**1**_; When *k =* 2, *y*_*2i*_ *=* 1, *y*_*1i*_ *= y*_*3i*_ *=* 0, the formula contains these points as *f*_*2*_; When *k =* 3, *y*_*3i*_ *=* 1, *y*_*1i*_ *= y*_*2i*_ *=* 0, the formula contains these points as *f*_*3*_, thus, the quadratic function that is:

$$f_{x}=y_{1i}f_{1}+y_{2i}f_{2}+y_{3i}f_{3}$$ (27)

Where *k* = 1,2,3; *i* = 1, 2, …, dim. Lagrange interpolation effectively utilizes the known data construct function to obtain the optimal value in the simplest form. Once the desired function is obtained, the optimal value is compared with that of the optimal individual of the original population. If the adaptive value of the optimal value of the optimal individual of the original population is better than that of the optimal individual of the original population, the individual is updated, and the next iteration is carried out.

### Newton interpolation PO (NIPO)

The function constructed by Newton interpolation method is similar to Lagrange, where each additional point does not need to be recalculated, only subsequent newly added points need to be calculated. The specific operation steps of the Newton interpolation method are as follows:

It assumes that there are three points (*x*_*1*_,*f*_*1*_), (*x*_*2*_,*f*_*2*_), (*x*_*3*_,*f*_*3*_). *x*_*1*_ = (*x*_*11*_,*x*_*12*_,*…*, *x*_*1i*_), *x*_*2*_ *=* (*x*_*21*_,*x*_*22*_,*…*,*x*_*2i*_), *x*_*3*_ = (*x*_*31*_,*x*_*32*_,*…*,*x*_*3i*_). *i* is the maximum dimension in this generation. The first order mean difference of Newton interpolation formula is obtained:

$$f\left[x_{1i},x_{2i}\right]=\frac{f\left(x_{1i}\right)-f\left(x_{2i}\right)}{x_{1i}-x_{2i}}$$ (28)

The second-order mean difference can be obtained after adding a point:

$$f\left[x_{1i},x_{2i},x_{3i}\right]=\frac{f\left[x_{1i},x_{2i}\right]-f\left[x_{2i},x_{3i}\right]}{x_{1i}-x_{3i}}$$ (29)

Therefore, the function of Newton interpolation method can be obtained as follows:

$$f\left(x_{i}\right)=f\left(x_{1i}\right)+f\left[x_{1i},x_{2i}\right]\left(x-x_{1i}\right)+f\left[x_{1i},x_{2i},x_{3i}\right]\left(x-x_{1i}\right)\left(x-x_{2i}\right)$$ (30)

Thus, we can find the optimal solution *Q* from the function. The optimal solution is compared with the optimal individual in the original population. If the fitness value *f(Q)* of the optimal solution is better than the fitness value of the original optimal individual, the optimal individual is updated. Otherwise, the optimal individual of the original population is retained, and the next iteration is carried out.

### Refraction learning

**Fig 6:**
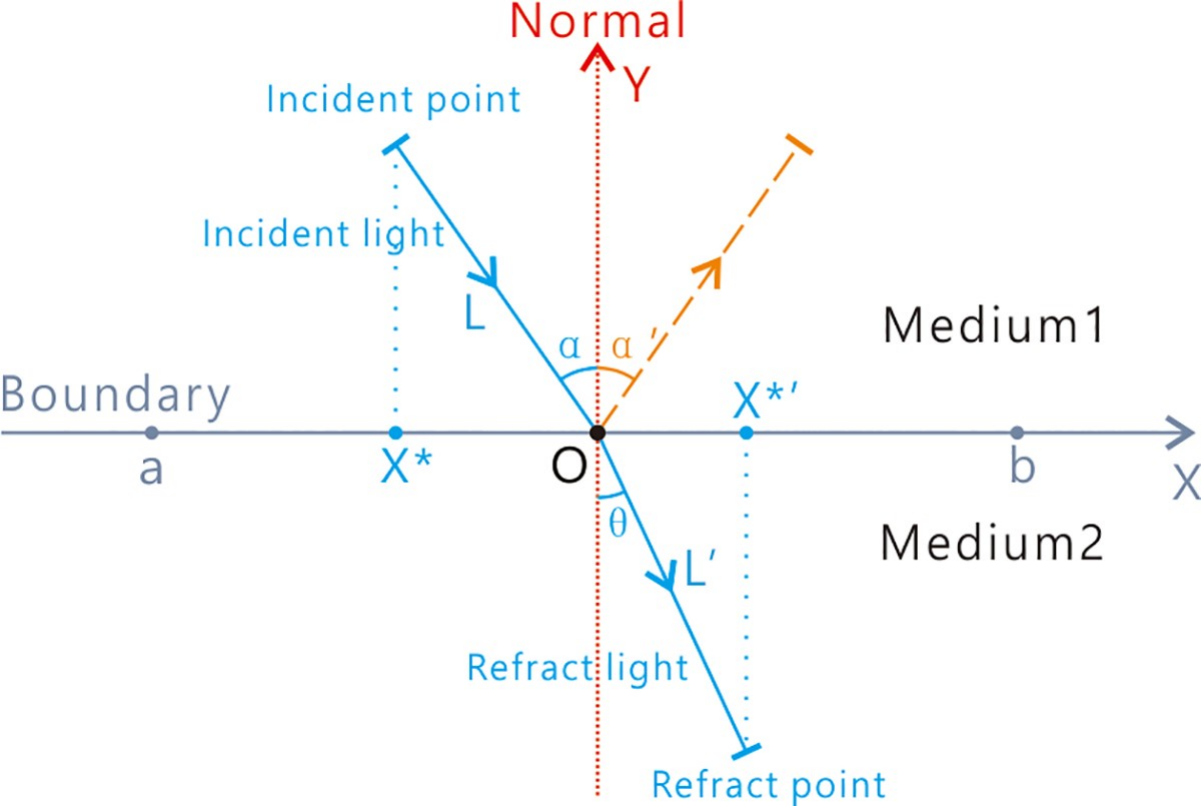
The principle of refraction learning.

If only the interpolation strategy is utilized, the diversity of the population will quickly decline. Therefore, a RL strategy was proposed to assist with finding a potentially better area and maintaining the diversity of PO. RL was proposed by Long (2019) [57, 58], which has a strong and broad searching capacity. As seen in Fig 6, the lower bound of the search is *a* and the upper bound of the search is *b*. Y indicates the normal. A is the incident point and B is the refraction point. The length of AO is *L* and that of BO is *L’*. α is the incident angle and *θ* is the refraction angle.

Based on Fig 6, we can calculate ***sin***(***α***) and ***sin***(***θ***) as follows:

$$sin\left(\alpha \right)=\frac{\left(\frac{a+b}{2}-X^{*}\right)}{L}$$ (31)

$$sin\left(\theta \right)=\frac{\left(X^{*\prime }-\frac{a+b}{2}\right)}{L^{\prime }}$$ (32)

From above two formulas, the refraction index *p* is obtained as:

$$p=\frac{sin\left(\alpha \right)}{sin\left(\theta \right)}=\frac{\left(\left(a+b\right)/2-X^{*}\right)/L}{\left(X^{*\prime }-\left(a+b\right)/2\right)/L^{\prime }}$$ (33)

Assuming $\xi =\frac{L}{L^{\prime }}$, the above formula is rewritten as:

$$\xi p=\frac{\left(\left(a+b\right)/2-X^{*}\right)}{\left(X^{*\prime }-\left(a+b\right)/2\right)}$$ (34)

The n-dimensional space obtains:

$$X_{j}^{*\prime }=\frac{a_{j}+b_{j}}{2}+\frac{a_{j}+b_{j}}{2\xi p}-\frac{X_{j}^{*}}{\xi p}$$ (35)

As shown in Fig 6, Y indicates the normal. A is the incident point and B is the refraction point. The length of AO is *L* and that of BO is *L’*. α is the incident angle and *θ* is the refraction angle. As shown in Eq (35), where $X_{j}^{*}$ is the jth dimension of solution X^*^; $X_{j}^{*\prime }$ is the jth dimension of the opposite solution X*′; *a*_*j*_ is the lower bound of the *j*_*th*_ dimension;*b*_*j*_ is the upper bound of the *j*_*th*_ dimension.

### Adaptive parameter based on logistic model

Obtaining a good balance between exploration and exploitation is the key component of optimization techniques. Global exploration involves the exploration of new potential areas. Consequently, it is critical to maintain the diversity of the population. Local exploitation involves searching for a high-precision solution in a small area, which is revealed through global exploration. Excessive global exploration leads to a slower convergence speed, whereas excessive local exploitation results in premature convergence.

According to Askari, adaptive parameter λ should be adopted to obtain an improved balance between exploration and exploitation during the party switching stage [53]. In canonical PO, the λ values are linearly decreased from one to zero. However, it is not appropriate to adopt a linear parameter strategy to reflect and simulate the actual nonlinear search process. As is well acknowledged, at the onset of iterative optimization, a robust global search capacity is required to examine as many potential areas as possible. At the conclusion of the iterative optimization, a strong localized search ability is required to improve accuracy. During the party switching stage in PO, the larger the λ value, the stronger of global exploration. For the early iteration, a larger λ value can produce a larger step, which leads to stronger global exploration. At this stage, the declining speed of λ needs to be larger. For the late iteration, a smaller λ value can produce a smaller step, which leads to stronger local exploitation. However, the smaller λ value leads to a poorer population diversity and results in a higher probability at local optimum. At this stage, we require population jumping out of local optimum; thus, the decline speed of λ needs to be smaller. Suppose the maximum of λ is λ_***max***_ and the minimum of lamuda is λ_***min***_. Assume the initial decline speed of λ is k, after which it gradually slows down during the entire iterative process. Such a changing law of λ conforms to the logistic model [59], as shown in Eq (36):

$$\{\begin{array}{l} \frac{d\lambda \left(t\right)}{dt}=k\cdot \left(1-\frac{\lambda \left(t\right)}{\lambda_{min}}\right)\cdot \lambda \left(t\right)\\ \lambda \left(0\right)=\lambda_{max} \end{array}$$ (36)

We can employ means of separation variables to solve Eq (36), after which adaptive parameter adjustment based on a logistic model is obtained, as shown in Eq (37):

$$\lambda \left(t\right)=\frac{\lambda_{min}}{1+\left(\frac{\lambda_{min}}{\lambda_{max}}-1\right)\cdot e^{-kt}}$$ (37)

where *t* is the iteration count, and k is the initial decline speed. Assuming that *t* equals zero in Eq (37), we can then obtain λ(0) = λ_***max***_. If *t* is allowed to approach infinity, then *λ*(∞) *= λ*_*min*_ is obtained.

**Fig 7:**
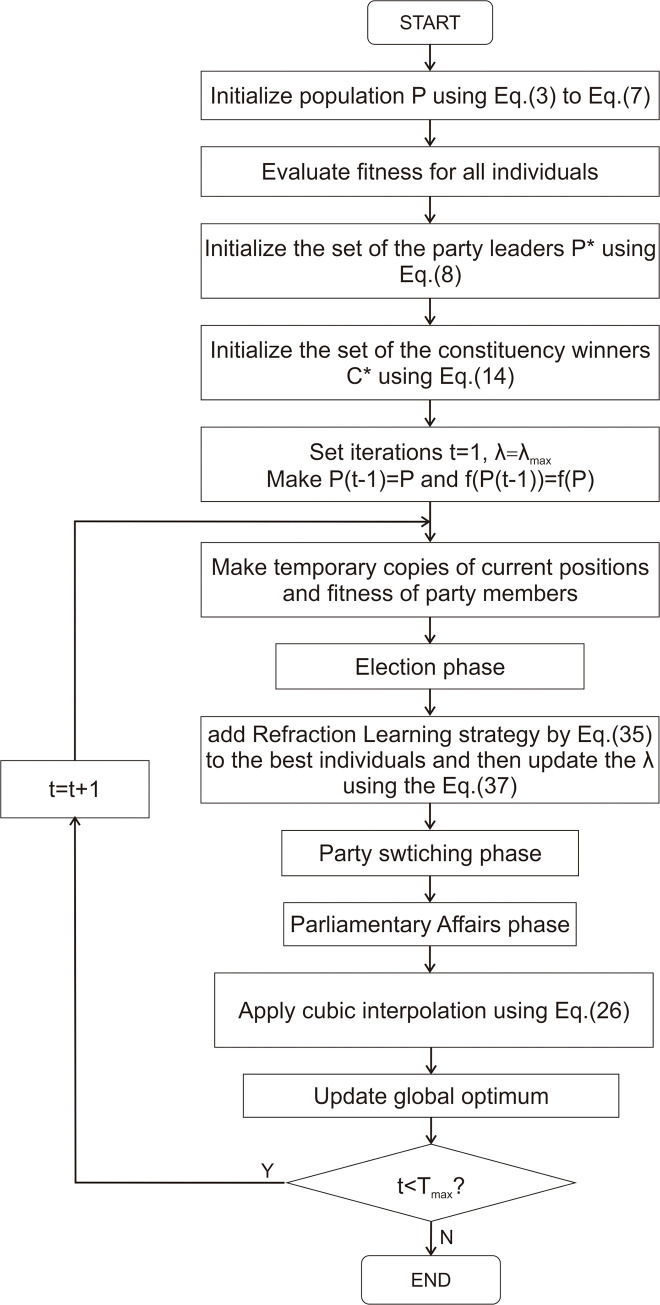
The flow chart of CRLPO.

Each of the above interpolation strategies is applied to PO. The fitness of individuals predicted by interpolation are compared with the fitness of optimal individuals obtained by the original PO. The optimal interpolation strategy is selected to combine with refraction strategy. The flow chart of the CRLPO algorithm is illustrated in Fig 7.

### Computation complexity

CRLPO preserves the canonical framework and main operations of PO, adding only RL with cubic interpolation following parliamentary affairs. Assuming the dimension of the objective function is D the population size is M and the maximum number of iterations is T. The main steps of CRLPO are election campaign, party switching, parliamentary affairs, and refraction learning. The time complexity of the election campaign is *O*(*TMD*). The time complexity of party switching is *O*(*TM*). The time complexity of parliamentary affairs is $O(T\sqrt{M}D)$. The time complexity of refraction learning, and cubic interpolation are both *O*(*TD*). The overall time complexity of CRLPO is $O(C\mathrm{RLPO})\approx O(TMD)+O(T\sqrt{M}D)+O(TM)+O(TD).$As the latter of above equation is a low order item, it can be omitted. Therefore, the overall time complexity of CRLPO is almost the same as that of the original PO. In summary, the improvement in PO does not add significant calculation costs.

## Results and analysis

### Benchmark and functions

To test the effectiveness of CRLPO, we benchmarked the CRLPO in this section with 19 classical and popular benchmark functions used by a many researchers [53, 60–64]. There were two categories of benchmark functions adopted: unimodal and multimodal.

As unimodal functions have no local optimum, they are quite suitable for the evaluation of exploitation capacities. Conversely, multimodal benchmark functions possess many local optimums and only one global optimum. Therefore, they are quite suitable for evaluating the exploration abilities of algorithms. The main characteristics of the test functions are listed as follows:

### Performance metrics

To evaluate the experimental results, several performance indicators were adopted in this paper, such as best fitness, worst fitness, mean fitness, and fitness variance. These performance indicators were defined as follows:

$$BestFitness=Min\left(ExpReslt_{i}\right),i=1\ldots NR$$ (38)

$$WorstFitness=Max\left(ExpReslt_{i}\right),i=1\ldots NR$$ (39)

$$MeanFitness=\frac{1}{NR}\overset{NR}{\underset{i=1}{\sum }}\left(ExpReslt_{i}\right)$$ (40)

$$std=\sqrt{\overset{NR}{\underset{i=1}{\sum }}\frac{{\left(ExpReslt_{i}-MeanFitness\right)}^{2}}{NR}}$$ (41)

Where *NR* indicates the total number of independently repeated experiments, *ExpReslt* indicates the output function fitness of each independent experiment, and *i* is the current count of repeated experiments.

### Comparative results of interpolation strategy on benchmark functions Table 1

In experiment 1, to compare all above interpolation strategies in Sect III, all of the methods with the interpolation strategy were set at the same parameters: the population size was 64 and the maximum number of iterations was 417.

**Table 1:** Main characteristics of 19 widely used benchmark functions.

Function type	Function name	Function formula	Search range	*f*_*min*_
**Unimodal Functions**	Sphere	$f_{1}(x)=\sum_{i=1}^{n}x_{i}^{2}$	[-100,100]	0
	Schwefel’s 2.22	$f_{2}(x)=\sum_{i=1}^{n}|x_{i}|+\prod_{i=1}^{n}\left|x_{i}\right|$	[-10,10]	0
	Rotated Hyper-ellipsoid	$f_{3}(x)=\sum_{i=1}^{n}(\sum_{j=1}^{i}x_{j}{)}^{2}$	[-100,100]	0
	Schwefel’s 2.21	$f_{4}(x)=\max_{i}\{|x_{i}|,1\leq i\leq n\}$	[-100,100]	0
	Rosenbrock	$f_{5}(x)=\sum_{i=1}^{n-1}\begin{array}{c} {}[100(x_{i+1}-x_{i}^{2}{)}^{2}\\ +(x_{i}-1{)}^{2}] \end{array}$	[-30,30]	0
	Step	$f_{6}(x)=\sum_{i=1}^{n}(|x_{i}+0.5|{)}^{2}$	[-100,100]	0
	Noise	$f_{7}(x)=\sum_{i=1}^{n}ix_{i}^{4}+random[0,1]$	[-1.28,1.28]	0
**Multi-modal Functions**	Schwefel’s 2.26	$f_{8}(x)=\sum_{i=1}^{n}-x_{i}\sin(\sqrt{\left|x_{i}\right|})$	[-500,500]	-418.9829*D
	Rastrigin	$f_{9}(x)=\sum_{i=1}^{n}\begin{array}{c} \left|x_{i}^{2}-10\;\cos(2\pi x_{i}\right)\\ +10| \end{array}$	[-5.12,5.12]	0
	Ackley	$f_{10}(x)=20+e-20\;\exp\left(-0.2\sqrt{\frac{1}{n}\sum_{i=1}^{n}x_{i}^{2}}\right)-\exp\left(\frac{1}{n}\sum_{i=1}^{n}\cos(2\pi x_{i})\right)$	[-32,32]	0
	Griewank	$f_{11}(x)=1+\frac{1}{4000}\sum_{i=1}^{n}x_{i}^{2}-\prod_{i=1}^{n}\cos(\frac{x_{i}}{\sqrt{i}})$	[-600,600]	0
	Pendlized	$f_{12}(x)=\frac{\pi }{n}\{10\;\sin(\pi y_{1})+\sum_{i=1}^{n-1}(y_{i}-1{)}^{2}[1+10\;\sin^{2}(\pi y_{i+1})+(y_{n}-1{)}^{2}]\}+\sum_{i=1}^{n}u(xi,10,100,4)$	[-50,50]	0
		$y_{i}=1+\frac{x_{i}+1}{4},u(x_{i},a,k,m)=\left\{ \begin{array}{l} k(x_{i}-a{)}^{m},x_{i}>a\\ 0,-a<x_{i}<a\\ k(-x_{i}-a{)}^{m},x_{i}<-a \end{array}\right.$		
	Gen. Pendlized	$f_{13}(x)=0.1\{\sin^{2}(3\pi x_{1})+\sum_{i=1}^{n}(x_{i}-1{)}^{2}[1+\sin^{2}(3\pi x_{i})]+(x_{n}-1{)}^{2}[1+\sin^{2}(2\pi x_{n})]\}+\sum_{i=1}^{n}u(x_{i},5,100,4)\;,u(x_{i},a,k,m)=\left\{ \begin{array}{l} k(x_{i}-a{)}^{m},x_{i}>a\\ 0,-a<x_{i}<a\\ k(-x_{i}-a{)}^{m},x_{i}<-a \end{array}\right.$	[-50,50]	0
	Periodic	$f_{14}(x)=1+\sum_{i=1}^{n}\sin^{2}\left(x_{i}\right)-0.1exp(-\sum_{i=1}^{n}x_{i}^{2})$	[-10,10]	0.9
	Alpine N.1	$f_{15}(x)=\sum_{i=1}^{n}\left(\left|x_{i}sinx_{i}+0.1x_{i}\right|\right)$	[-10,10]	0
	Xin-She Yang	$f_{16}(x)=f_{16}(x)+rand(|x_{i}^{i}|)$	[-5,5]	0
	Salomon	$f_{17}(x)=1-\cos\left(2\pi \sqrt{\sum_{i=1}^{n}x_{i}^{2}}\right)+0.1\sqrt{\sum_{i=1}^{n}x_{i}^{2}}$	[-100,100]	0
	Styblinski-Tang	$f_{18}(x)=0.5(f_{18}(x)+x_{i}^{4}-16x_{i}^{2}+5x_{i})$	[-5,5]	-39.1659*D
	Xin-She Yang N.4	$f_{19}(x)=\left(\sum_{i=1}^{n}\left|x_{i}\right|-\exp\left(-\sum_{i=1}^{n}x^{2}\right)\right)exp(-\sum_{i=1}^{n}sin(x_{i}^{2}))$	[-10,10]	-1

**Fig 8:**
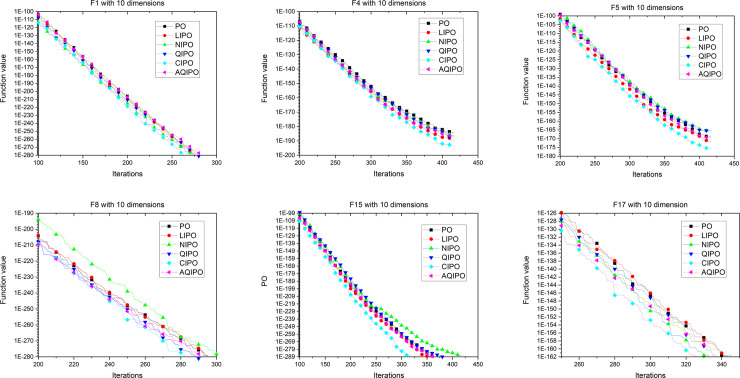
Convergence curve on 6 representative benchmark functions with 10 dimensions.

**Fig 9:**
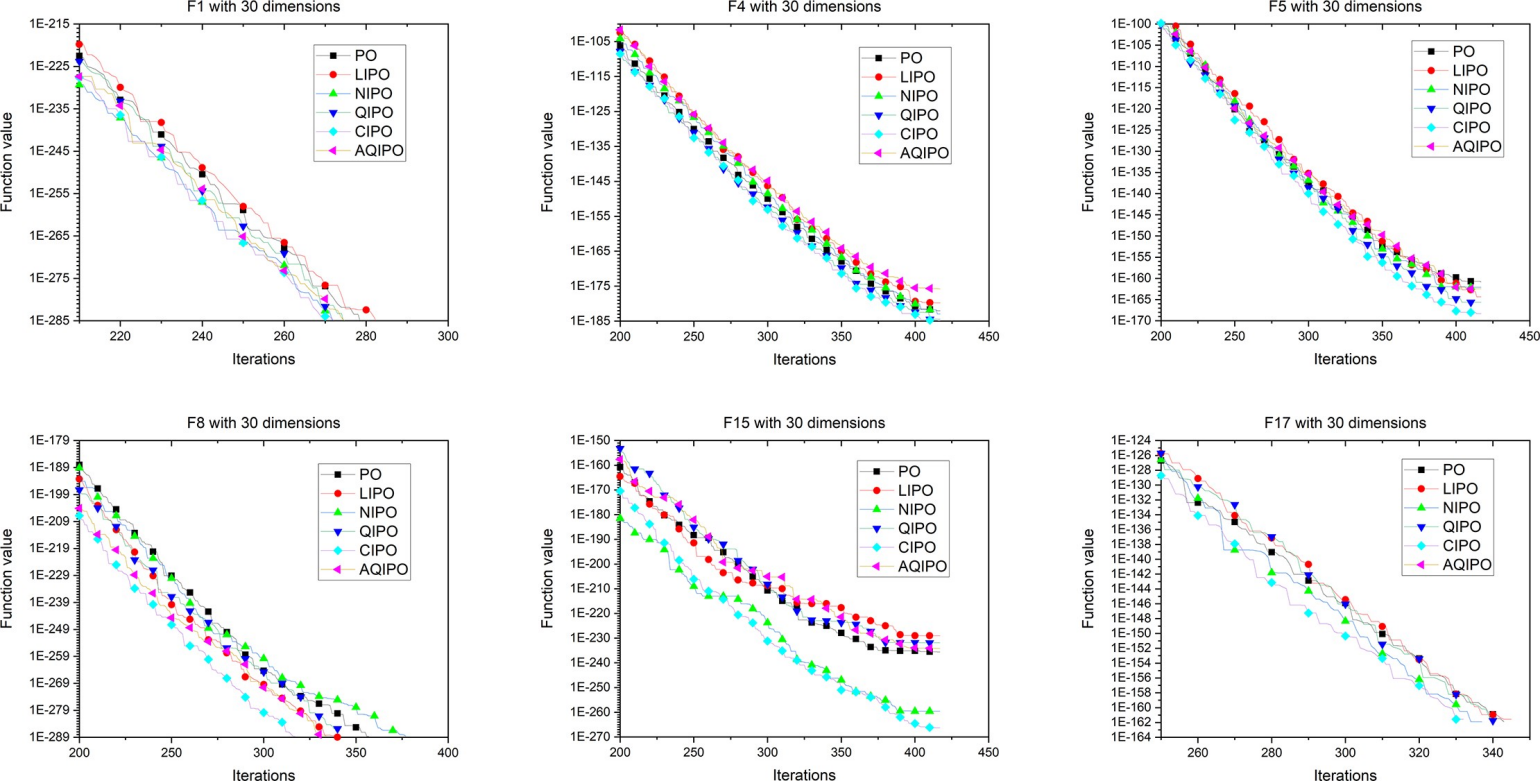
Convergence curve on 6 representative benchmark functions with 30 dimensions.

**Fig 10:**
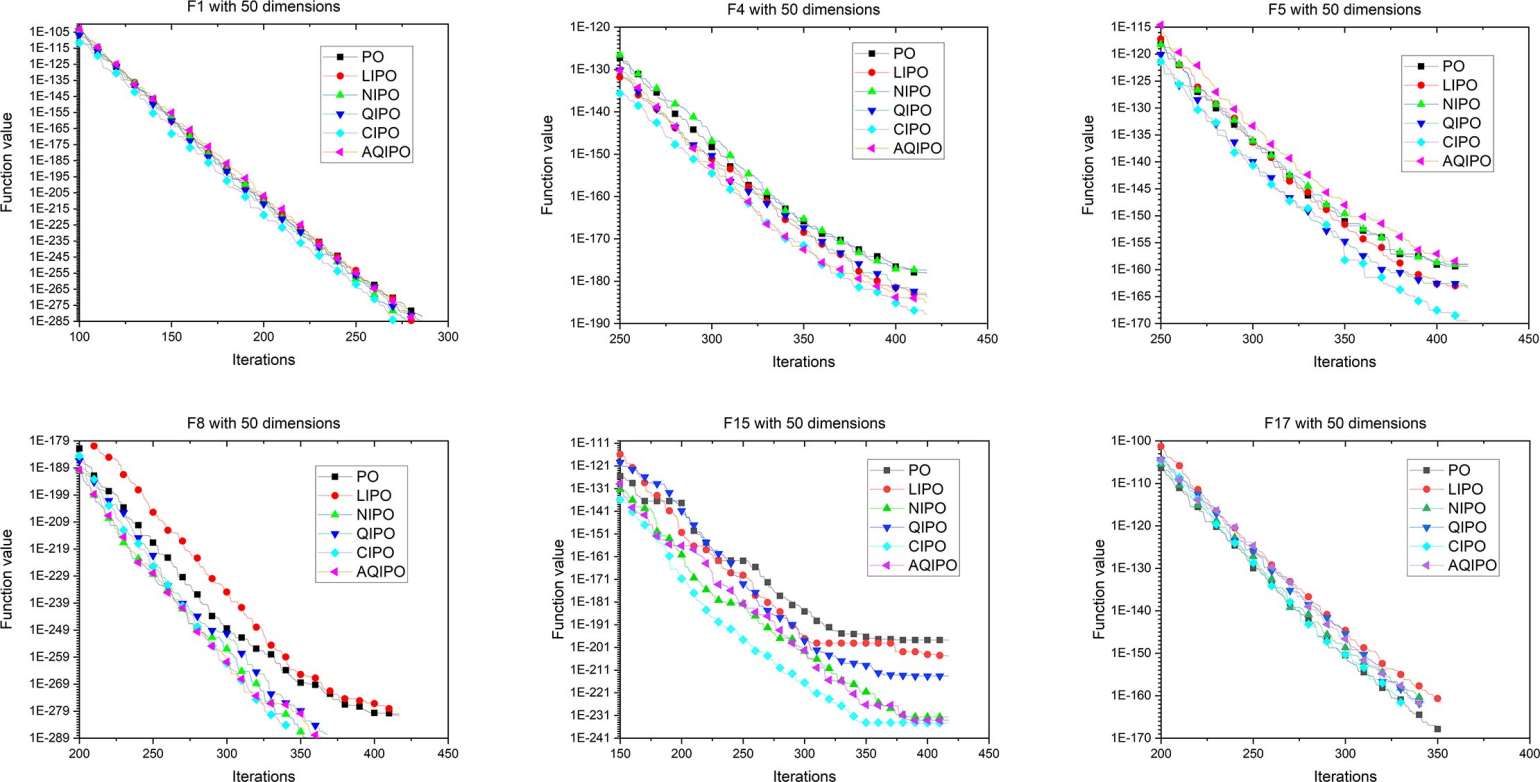
Convergence curve on 6 representative benchmark functions with 50 dimensions.

Fig 8 shows the convergence process of different interpolation strategies applied in PO with 10 dimensions. To further verify the performance, interpolation strategies are applied in PO with 30 and 50 dimensions. The convergence curves with 30 and 50 dimensions are depicted in Figs 9 and 10, respectively, which show the convergence processes of various interpolation strategies applied to the PO algorithm. The experimental results revealed the same conclusion; cubic interpolation has better performance than other interpolation strategies in different dimensions.

If interpolation strategy is utilized solely, the diversity of the population quickly declines; therefore, RL strategy was adopted to improve the diversity of the population.

Thus, on the basis of PO with the cubic interpolation strategy (CIPO), the RL strategy was applied into the CIPO, and we can assume that it enhances the performance of the algorithm. The CIPO algorithm with RL is referred to as CRLPO.

**Fig 11:**
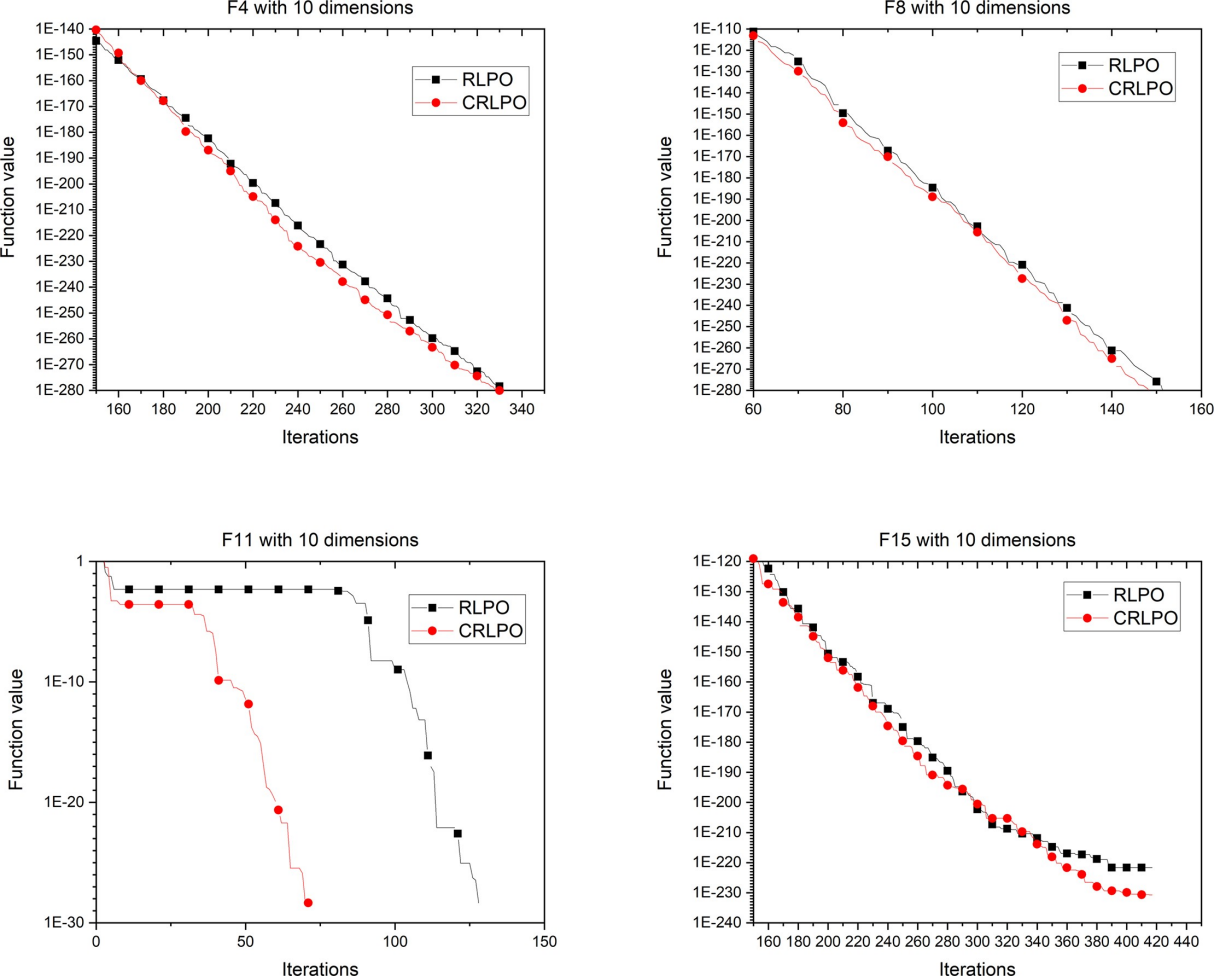
Convergence graph of CRLPO on 4 representative benchmark functions with 10 dimensions.

**Fig 12:**
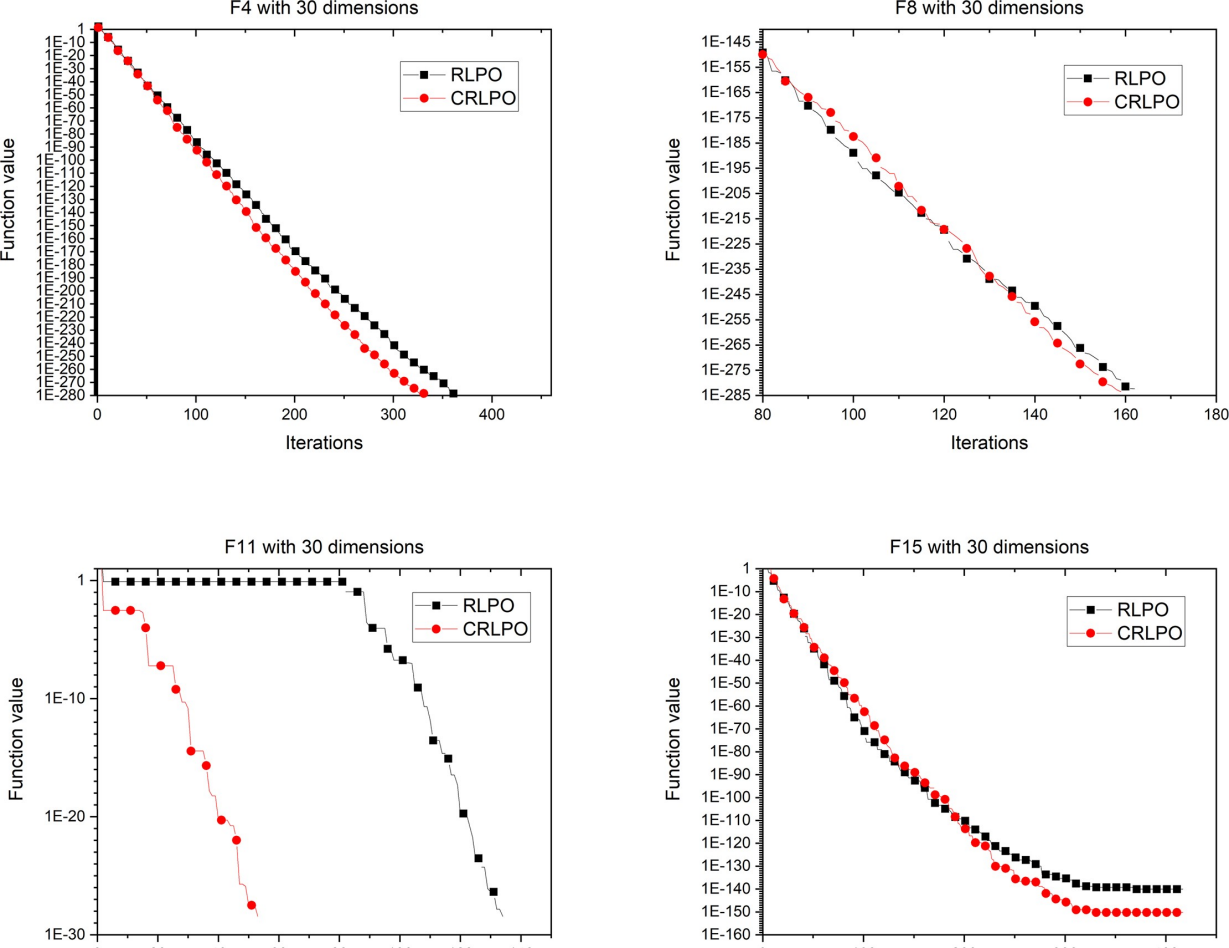
Convergence graph of CRLPO on 4 representative benchmark functions with 30 dimensions.

**Fig 13:**
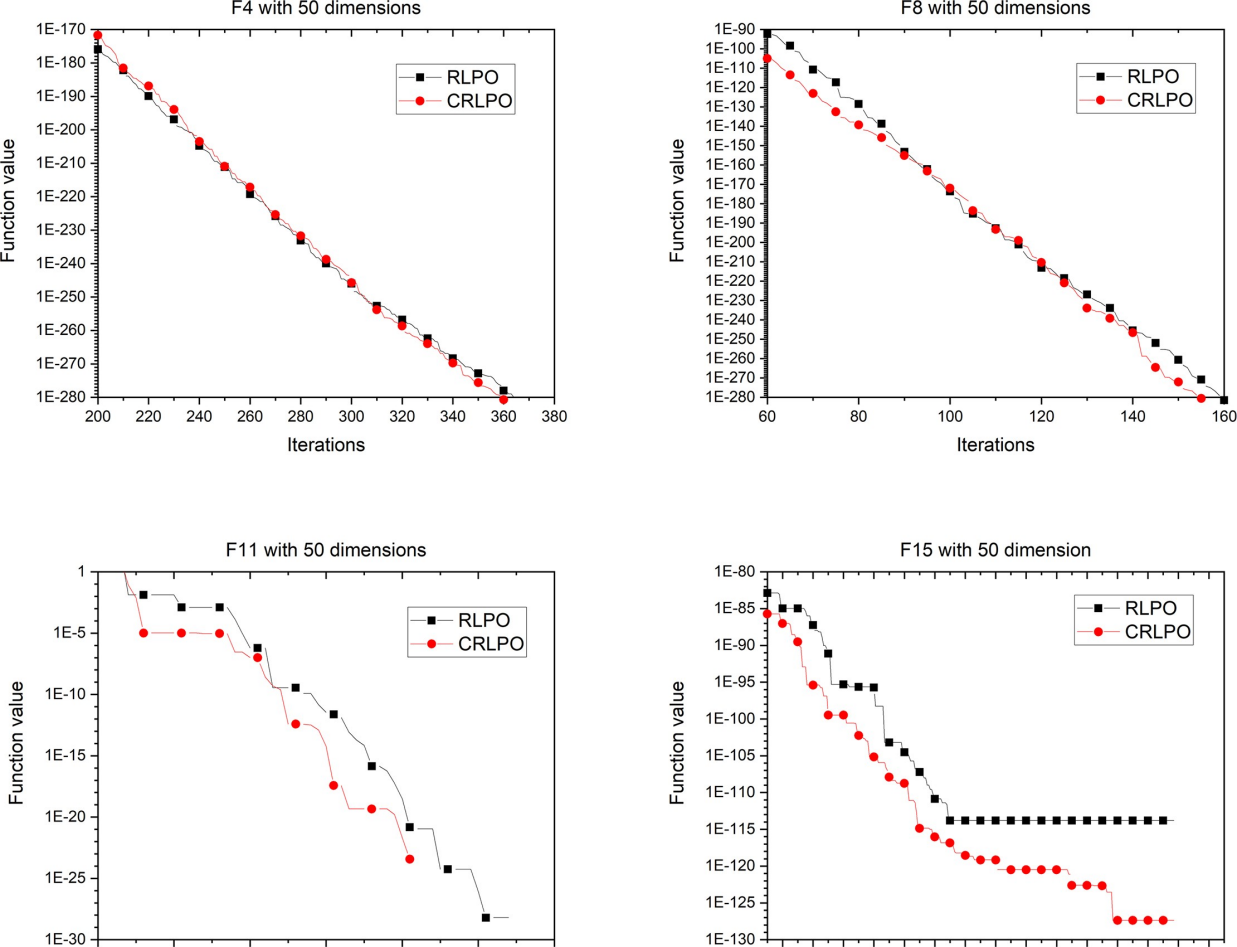
Convergence graph of CRLPO on 4 representative benchmark functions with 50 dimensions.

Experiments were conducted to compare the differences between RLPO and CRLPO under the same circumstances. As shown in Fig 11, the curve with square pattern represents the iteration of the PO algorithm using the RL strategy. It can be seen from Fig 11 that the convergence accuracy of the results were improved. For example, the RLPO algorithm requires 140 iterations to attain the convergence point with function 11, while following the addition of the cubic interpolation strategy, it requires only 50 iterations to reach the convergence point. Consequently, the cubic interpolation can improve the performance of the RLPO. The same results can be found in dimension 30 and dimension 50 as shown in Figs 12 and 13.

The above experimental results proved that the performance of CRLPO was better than that of RLPO. In the next section, CRLPO will be compared with other algorithms to evaluate its performance.

### Experiments on test functions: Table 1

For the sake of evaluating the performance of CRLPO, it and other state-of-the-art algorithms were run thirty times on each benchmark function with 30, 100, and 1000 dimensions from Table 1. The CRLPO was compared with eight algorithms, including PO [53], GWO [4], HHO [15], PSO [5], SCA [41], DE [25], WOA [2], and RLGWO [58].

For the purpose of fairness, we set the same common parameters for all algorithms: population size was 64 and the maximum number of iterations was 417. As to other specific parameters of all algorithms, we set them as follows: for the PO algorithm, the party number n = 8; for the GWO algorithm, parameter a was linearly decreased between two and zero; for the HHO algorithm, parameter E1 was linearly decreased between two and zero; for the PSO algorithm, wMax = 0.9; wMin = 0.2, c1 = 2, c2 = 2; in SCA algorithm, parameter r1 was linearly decreased between two and zero; for the DE algorithm, F = 0.5, Cr = 0.01; for the WOA algorithm, parameter a was linearly decreased between two and zero, and parameter a2 was linearly decreased between -1 and -2; for the RL-GWO algorithm, p = 100, ξ = 1000; for the CRLPO algorithm, party number n = 8, p = 100, ξ = 1000;

In experiment 2, CRLPO and other eight algorithms were run independently thirty times on 19 benchmark functions from Table 1 with 30 dimensions. Table 2 summarizes the average (Ave) and standard deviation (Std), and the results of the Friedman’s test (Ave.R denotes average rank and Ova.R represents overall rank). Table 3 summarizes the p-values of the Wilcoxon rank-sum test at a 0.05 significance level for CRLPO against the other eight algorithms.

**Table 2:** Experimental results of 19 benchmark functions from Table 1 with 30 dimensions.

F	Results	PO	PSO	DE	GWO	WOA	SCA	HHO	RLGWO	CRLPO
f1	Ave	0.00E+00	3.90E-05	3.17E+03	1.14E-29	6.01E-75	9.50E+00	2.51E-89	0.00E+00	0.00E+00
	Std	0.00E+00	3.31E-05	5.78E+02	1.60E-29	1.98E-74	2.29E+01	1.17E-88	0.00E+00	0.00E+00
f2	Ave	1.14E-179	2.34E+00	2.11E+01	1.25E-17	9.54E-47	1.61E-02	5.51E-45	0.00E+00	0.00E+00
	Std	0.00E+00	5.04E+00	2.48E+00	7.89E-18	1.68E-46	1.87E-02	2.13E-44	0.00E+00	0.00E+00
f3	Ave	9.79E-280	4.91E+01	4.11E+04	4.88E-07	2.84E+04	6.64E+03	9.12E-73	0.00E+00	0.00E+00
	Std	0.00E+00	1.48E+01	4.64E+03	1.17E-06	1.06E+04	3.99E+03	4.14E-44	0.00E+00	0.00E+00
f4	Ave	5.74E-159	8.32E-01	4.56E+00	1.88E-07	3.44E+01	2.64E+01	1.00E-44	0.00E+00	0.00E+00
	Std	2.99E-158	1.82E-01	9.04E+05	1.60E-07	2.80E+01	9.18E+00	3.26E-44	0.00E+00	0.00E+00
f5	Ave	8.95E-01	7.14E+01	1.13E+06	2.66E+01	2.74E+01	1.81E+04	4.75E-03	2.70E+01	0.00E+00
	Std	4.90E+00	4.91E+01	4.88E+05	7.36E-01	3.45E-01	4.95E+04	5.30E-03	9.19E-01	0.00E+00
f6	Ave	0.00E+00	2.58E-05	2.98E+03	3.10E-01	4.79E-02	9.51E+00	4.68E-05	4.79E-01	0.00E+00
	Std	0.00E+00	2.47E-05	6.59E+02	2.55E-01	5.7678–02	8.06E+00	7.02E-05	2.83E-01	0.00E+00
f7	Ave	2.77E-04	3.23E+00	1.39E+00	1.25E-03	2.57E-03	7.04E-02	6.29E-05	4.58E-04	4.30E-04
	Std	1.43E-04	4.16E+00	2.71E-01	5.45E-04	2.75E-03	6.66E-02	5.75E-05	3.73E-04	0.00E+00
f8	Ave	-1.23E+04	-5.94E+03	-7.58E+03	-6.37E+03	-1.08E+04	-3.91E+03	‐1.26E+04	-6.19E+03	‐1.26E+04
	Std	9.01E+02	1.29E+03	3.05E+02	6.55E+02	1.60E+03	2.58E+02	7.00E-01	6.45E+02	0.00E+00
f9	Ave	0.00E+00	9.04E+01	1.35E+02	3.07E+00	0.00E+00	2.64E+01	0.00E+00	0.00E+00	0.00E+00
	Std	0.00E+00	2.77E+01	1.46E+01	4.32E+00	0.00E+00	2.58E+01	0.00E+00	0.00E+00	0.00E+00
f10	Ave	8.88E‐16	6.98E-03	1.25E+01	7.05E-14	4.32E-15	1.21E+01	8.88E‐16	8.88E‐16	8.88E‐16
	Std	0.00E+00	8.57E-03	8.30E-01	6.57E-15	2.87E-15	9.24E+00	0.00E+00	0.00E+00	0.00E+00
f11	Ave	0.00E+00	4.93E-03	2.81E+01	2.07E-03	8.92E-03	9.68E-01	0.00E+00	0.00E+00	0.00E+00
	Std	0.00E+00	5.78E-03	5.82E+00	4.79E-03	2.75E-02	2.36E-01	0.00E+00	0.00E+00	0.00E+00
f12	Ave	1.57E‐32	3.46E-03	2.42E+04	2.39E-02	7.24E-03	2.76E+04	3.62E-06	2.40E-02	1.57E‐32
	Std	5.56E-48	1.89E-02	3.87E+04	1.46E-02	9.95E-03	1.05E+05	4.55E-06	1.49E-02	0.00E+00
f13	Ave	1.34E‐32	4.05E-03	5.56E+05	2.85E-01	1.70E-01	1.92E+04	3.75E-05	4.69E-01	1.34E‐32
	Std	5.56E-48	5.37E-03	4.03E+05	1.57E-01	1.39E-01	5.82E+04	8.31E-05	2.65E-01	0.00E+00
f14	Ave	9.40E-01	1.69E+01	1.42E+01	4.56E+00	1.10E+00	6.09E+00	9.00E‐01	9.00E‐01	9.00E‐01
	Std	4.98E-02	4.88E+00	6.78E-01	5.75E+00	5.98E-01	1.23E+00	4.52E-16	4.52E-16	9.00E-01
f15	Ave	3.70E-16	2.68E+01	7.97E+01	4.26E-03	6.00E-46	1.39E+00	3.37E-47	0.00E+00	0.00E+00
	Std	2.02E-15	7.56E+00	4.22E+00	1.92E-03	2.74E-45	2.25E+00	9.78E-47	0.00E+00	0.00E+00
f16	Ave	4.07E-193	2.57E+44	2.78E+48	4.39E-12	3.69E-03	6.83E-01	5.55E-11	0.00E+00	0.00E+00
	Std	0.00E+00	1.40E+45	1.52E+49	2.41E-11	1.18E-02	2.32E+00	3.77E+04	0.00E+00	0.00E+00
f17	Ave	6.65E-03	1.68E+00	3.55E+01	3.50E-01	1.23E-01	9.16E-01	1.15E-43	0.00E+00	0.00E+00
	Std	2.53E-02	1.14E-01	1.52E+00	5.57-E02	7.74E-02	5.26E-01	4.73E-43	0.00E+00	0.00E+00
f18	Ave	-1.96E+03	-3.23E+03	-2.82E+03	-2.42E+03	-3.77E+03	-6.18E+02	-1.17E+03	-9.53E+02	-1.17E+03
	Std	4.62E-13	9.53E+01	4.59E+01	1.05E+02	2.35E+02	2.79E+01	2.27E-03	5.46E+01	0.00E+00
f19	Ave	-7.00E-01	6.05E-41	1.05E-37	2.49E-41	-2.00E-01	2.50E-10	-1.00E+00	-1.00E+00	-1.00E+00
	Std	4.66E-01	1.71E-40	1.29E-37	4.34E-41	4.07E-01	1.58E-10	0.00E+00	0.00E+00	0.00E+00
**Ave.R**		**2.63**	**6.58**	**8.11**	**5.47**	**5.05**	**7.84**	**2.79**	**3.00**	**1.42**
**Ova.R**		**2**	**7**	**9**	**6**	**5**	**8**	**3**	**4**	**1**

**Table 3:** p-values of the Wilconxon rank-sum test at 0.05 significance level for CRLPO against other eight algorithms on 19 benchmark functions from Table 1 with 30 dimensions.

F	PO	DE	PSO	WOA	GWO	SCA	HHO	RLGWO
	P-value	P-value	P-value	P-value	P-value	P-value	P-value	P-value
f1	NAN	1.78E-06	1.78E-06	1.78E-06	1.78E-06	1.78E-06	1.78E-06	NAN
f2	1.78E-06	1.78E-06	1.78E-06	1.78E-06	1.78E-06	1.78E-06	1.78E-06	NAN
f3	3.90E-06	1.78E-06	1.78E-06	1.78E-06	1.78E-06	1.78E-06	1.78E-06	NAN
f4	1.78E-06	1.78E-06	1.78E-06	1.78E-06	1.78E-06	1.78E-06	1.78E-06	NAN
f5	NAN	1.78E-06	1.78E-06	1.78E-06	1.78E-06	1.77E-06	1.78E-06	1.78E-06
f6	NAN	1.78E-06	1.78E-06	1.78E-06	1.78E-06	1.78E-06	1.78E-06	1.78E-06
f7	3.92E-02	1.78E-06	1.78E-06	3.60E-06	1.78E-06	2.27E-04	1.78E-06	6.27E-04
f8	NAN	1.78E-06	1.78E-06	2.59E-04	1.78E-06	NAN	1.77E-06	1.77E-06
f9	NAN	1.78E-06	1.78E-06	NAN	1.67E-06	NAN	1.78E-06	NAN
f10	NAN	1.78E-06	1.77E-06	5.53E-05	1.52E-06	NAN	1.78E-06	NAN
f11	NAN	1.78E-06	1.65E-06	NAN	NAN	NAN	1.78E-06	NAN
f12	NAN	1.78E-06	1.78E-06	1.77E-06	1.78E-06	1.78E-06	1.78E-06	1.78E-06
f13	NAN	1.78E-06	1.55E-06	1.78E-06	1.78E-06	1.78E-06	1.78E-06	1.78E-06
f14	4.88E-04	1.78E-06	1.78E-06	3.13E-02	1.78E-06	NAN	1.78E-06	NAN
f15	3.07E-04	1.78E-06	1.78E-06	1.78E-06	1.77E-06	1.78E-06	1.78E-06	NAN
f16	1.78E-06	1.78E-06	1.78E-06	1.78E-06	1.78E-06	1.78E-06	1.78E-06	NAN
f17	NAN	1.78E-06	1.71E-06	1.00E-06	8.90E-07	1.78E-06	1.78E-06	NAN
f18	4.47E-08	1.74E-06	1.77E-06	1.53E-06	1.76E-06	NAN	1.78E-06	1.78E-06
f19	3.91E-03	4.47E-08	4.47E-08	1.00E-06	4.47E-08	NAN	1.78E-06	NAN
**+**	**9**	**19**	**19**	**17**	**18**	**12**	**19**	**7**
**-**	**0**	**0**	**0**	**0**	**0**	**0**	**0**	**0**
**≈**	**10**	**0**	**0**	**2**	**1**	**7**	**0**	**12**

According to Table 2, CRLPO outperformed PO, PSO, DE, and GWO on 2, 3, 4, and 5 benchmark test functions, respectively. Furthermore, CRLPO was similar to PO on 1, 9, 10, 11, 12, and 13 benchmark test functions. With respect to WOA, SCA, HHO and RLGWO, CRLPO found better results than these four algorithms on 6, 7, 12, and 13 benchmark test functions, respectively. In addition, CRLPO was similar to HHO and RLGWO on 14 and 19 benchmark test functions, respectively. However, it was surpassed by HHO, PO, and WOA in only one case. Additionally, CRLPO was similar to WOA and HHO on 18 and 19 benchmark test functions, respectively. According to Table 2, CRLPO did the best on the Friedman’s test.

As is well known, the Wilconxon rank-sum test is one of the most frequently used statistical significance analyses; therefore, it was employed to evaluate the performance of CRLPO. As shown in Table 3, the results of a pair-wise comparison of CRLPO and other algorithms were demonstrated at a 0.05 significance level with 30 dimensions. In Table 3,“+” represents the number of functions in CRLPO that significantly outperformed an optimization technique, “-” indicates the number of functions where CRLPO was statistically surpassed by an optimization technique, and “≈” denotes the number of CRLPO functions that were similar to an algorithm. As illustrated in Table 3, the larger number in the “+” field and the lower number in “-” field revealed that CRLPO was statistically significant and relatively better than other optimization techniques.

**Fig 14:**
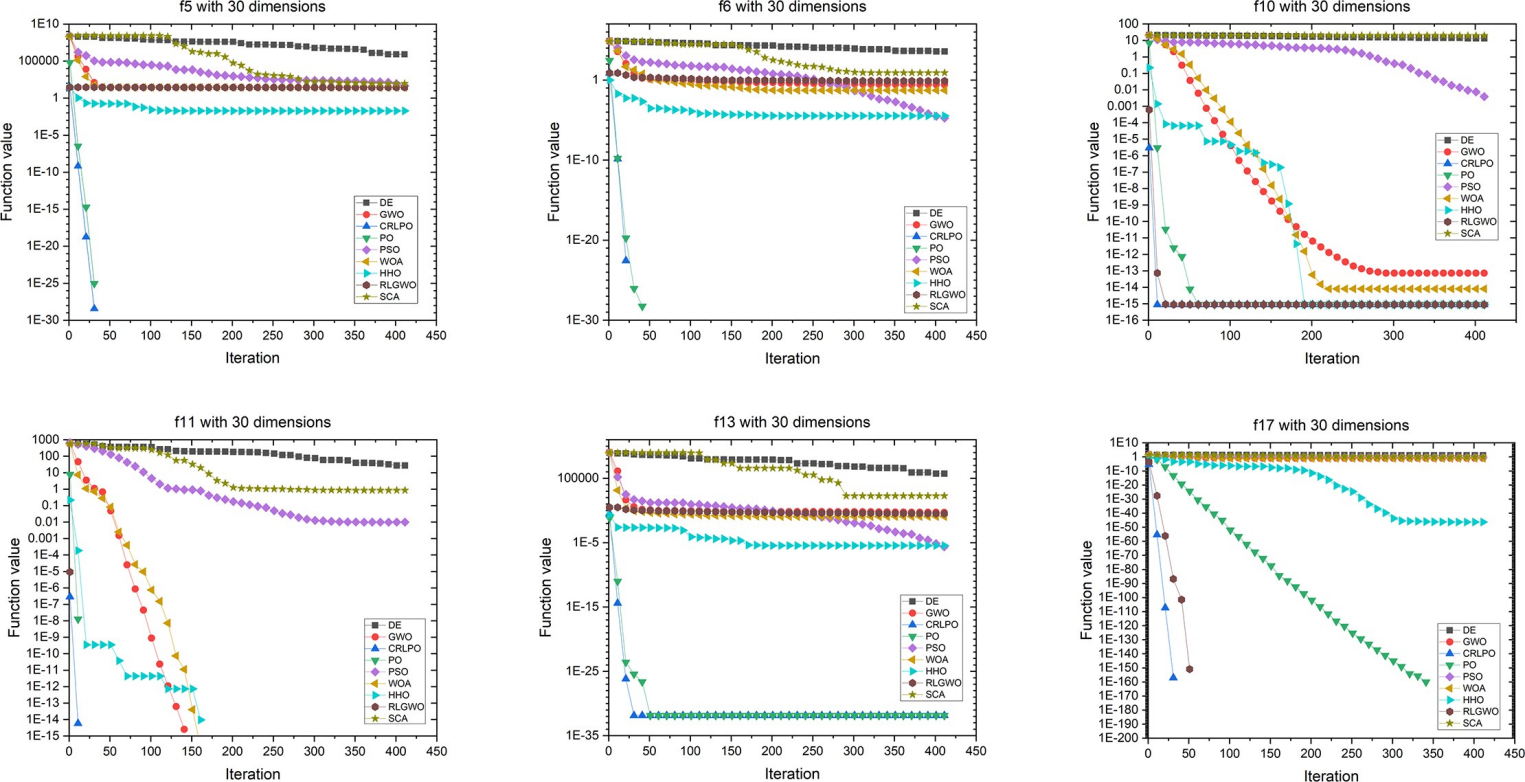
Convergence graph of CRLPO and eight other algorithms on six representative benchmark functions with 30 dimensions.

Fig 14 demonstrates the evolutionary process of the mean of the optimal value on 6 representative benchmark functions with 30 dimensions. As shown in Fig 14, CRLPO converged faster than did the other eight optimization techniques for all six representative cases. To ulteriorly observe and study the scalability of CRLPO, the proposed algorithm was tested with problems with higher dimensions. In experiment 3 CRLPO and the eight other algorithms were run independently 30 times on 19 benchmark functions (Table 1) with 100 dimensions. Table 4 summarizes the average (Ave) and standard deviation (Std) and the results of the Friedman’s test with 100 dimensions. Table 5 summarizes the p-values of the Wilconxon rank-sum test at a 0.05 significance level for CRLPO against the other eight algorithms with 100 dimensions.

**Table 4:** Experimental results of 19 benchmark functions from Table 1 with 100 dimensions.

F	Results	PO	PSO	DE	GWO	WOA	SCA	HHO	RLGWO	CRLPO
f1	Ave	0.00E+00	2.50E-05	3.40E+04	3.39E-29	1.62E-70	1.06E+04	6.04E-84	0.00E+00	0.00E+00
	Std	0.00E+00	2.20E-05	3.09E+03	6.45E-29	8.84E-70	7.29E+03	3.22E-83	0.00E+00	0.00E+00
f2	Ave	4.26E-178	2.01E+00	1.34E+02	1.01E-17	3.39E-45	7.57E+00	2.69E-65	0.00E+00	0.00E+00
	Std	0.00E+00	4.84E+00	7.36E+00	6.39E-18	1.50E-44	6.37E+00	1.46E-43	0.00E+00	0.00E+00
f3	Ave	1.52E-241	4.85E+01	2.98E+05	1.00E-06	8.59E+05	2.49E+05	1.05E-65	0.00E+00	0.00E+00
	Std	0.00E+00	1.49E+01	2.89E+04	3.99E-06	2.30E+05	6.08E+04	5.49E-65	0.00E+00	0.00E+00
f4	Ave	4.59E-156	8.47E-01	8.49E+01	1.94E-07	7.32E+01	8.97E+01	1.17E-42	0.00E+00	0.00E+00
	Std	1.99E-155	1.98E-01	1.83E+00	1.98E-07	2.28E+01	2.04E+00	4.83E-42	0.00E+00	0.00E+00
f5	Ave	0.00E+00	8.49E+01	3.47E+07	2.67E+01	9.78E+01	1.32E+08	1.16E-02	9.76E+01	0.00E+00
	Std	0.00E+00	8.97E+01	7.33E+06	7.82E-01	3.15E-01	6.09E+07	1.90E-02	7.74E-01	0.00E+00
f6	Ave	0.00E+00	5.28E-05	3.36E+04	4.17E-01	1.49E+00	9.78E+03	1.01E-04	8.31E+00	0.00E+00
	Std	0.00E+00	9.11E-05	3.21E+03	3.44E-01	5.13E-01	6.65E+03	1.69E-04	7.73E-01	0.00E+00
f7	Ave	2.77E-04	1.53E+00	5.05E+01	1.46E-03	2.23E-03	1.27E+02	7.81E-05	6.62E-04	2.97E‐05
	Std	2.40E-04	3.36E+00	1.31E+01	5.95E-04	2.45E-03	5.14E+01	7.33E-05	6.14E-04	0.00E+00
f8	Ave	-4.15E+04	-5.86E+03	-2.01E+04	-6.17E+03	-3.81E+04	-7.12E.+03	-4.19E+04	-1.64E+04	-4.19E+04
	Std	2.16E+03	1.27E+03	5.62E+02	5.74E+02	4.57E+03	5.56E+02	9.29E-01	3.02E+03	0.00E+00
f9	Ave	0.00E+00	9.15E+01	7.02E+02	2.60E+00	0.00E+00	2.32E+02	0.00E+00	0.00E+00	0.00E+00
	Std	0.00E+00	3.16E+01	1.64E+01	3.83E+00	0.00E+00	8.47E+01	0.00E+00	0.00E+00	0.00E+00
f10	Ave	8.88E‐16	3.03E-02	1.64E+01	6.86E-14	4.91E-15	1.84E+01	8.88E‐16	8.88E‐16	8.88E‐16
	Std	0.00E+00	1.20E-01	3.39E-01	7.34E-15	2.42E-15	4.58E+00	0.00E+00	0.00E+00	0.00E+00
f11	Ave	0.00E+00	1.48E-02	3.13E+02	2.15E-03	0.00E+00	1.05E+02	0.00E+00	0.00E+00	0.00E+00
	Std	0.00E+00	1.63E-02	2.45E+01	5.10E-03	0.00E+00	4.47E+01	0.00E+00	0.00E+00	0.00E+00
f12	Ave	4.71E‐33	7.01E-07	2.73E+07	2.35E-02	1.45E-02	2.81E+08	9.62E-07	1.63E-01	4.71E‐33
	Std	1.39E-48	1.06E-06	1.17E+07	1.15E-02	4.52E-03	1.27E+08	1.42E-06	3.85E-02	0.00E+00
f13	Ave	1.35E‐32	2.95E-03	9.37E+07	3.15E-01	1.12E+00	5.24E+08	4.13E-05	8.76E+00	1.35E‐32
	Std	5.57E-48	4.94E-03	2.41E+07	1.17E-01	5.02E-01	2.04E+08	4.25E-05	4.88E-01	0.00E+00
f14	Ave	9.50E-01	1.71E+01	1.42E+01	4.26E+00	1.45E+00	2.88E+01	9.00E‐01	9.00E‐01	9.00E‐01
	Std	5.09E-02	4.90E+00	7.93E-01	5.65E+00	1.33E+00	2.51E+00	4.52E-16	4.52E-16	0.00E+00
f15	Ave	5.55E-179	2.85E+01	7.89E+01	3.73E-03	1.48E-45	2.38E+01	5.90E-46	0.00E+00	0.00E+00
	Std	0.00E+00	7.46E+00	5.64E+00	2.48E-03	7.62E-45	1.29E+01	2.22E-45	0.00E+00	0.00E+00
f16	Ave	6.79E-195	5.31E+41	1.32E+47	3.48E-17	3.52E-03	2.91E+35	2.75E-18	0.00E+00	0.00E+00
	Std	0.00E+00	2.01E+42	4.62E+47	1.89E-16	1.62E-02	1.43E+36	1.51E-17	0.00E+00	0.00E+00
f17	Ave	3.33E-03	1.64E+00	3.57E+01	3.33E-01	1.27E-01	1.08E+01	1.50E-44	0.00E+00	0.00E+00
	Std	1.82E-02	1.38E-01	1.37E+00	6.06E-02	6.40E-02	3.40E+00	4.26E-44	0.00E+00	0.00E+00
f18	Ave	-3.87E+03	-3.18E+03	-2.82E+03	-2.42E+03	-3.70E+03	-1.49E+03	‐3.92E+03	-2.44E+03	‐3.92E+03
	Std	2.58E+02	1.13E+02	4.91E+01	1.26E+02	3.34E+02	8.85E+01	1.17E-02	1.42E+02	0.00E+00
f19	Ave	-7.00E-01	1.25E-40	1.23E-37	2.94E-41	-3.33E-01	5.03E-29	‐1.00E+00	‐1.00E+00	‐1.00E+00
	Std	4.66E-01	1.89E-40	1.13E-37	1.03E-40	4.79E-01	1.10E-28	0.00E+00	0.00E+00	0.00E+00
**Ave.R**		**2.32**	**6.37**	**8.11**	**5.79**	**5.11**	**7.89**	**3.00**	**2.95**	**1.00**
**Ova.R**		**2**	**7**	**9**	**6**	**5**	**8**	**4**	**3**	**1**

**Table 5:** p-values of the Wilconxon rank-sum test at 0.05 significance level for CRLPO against other eight algorithms on 19 benchmark functions from Table 1 with 100 dimensions.

Function	PO	DE	PSO	WOA	GWO	SCA	HHO	RLGWO
	P-value	P-value	P-value	P-value	P-value	P-value	P-value	P-value
f1	NAN	1.78E-06	1.78E-06	1.78E-06	1.78E-06	1.78E-06	1.78E-06	NAN
f2	1.78E-06	1.78E-06	1.78E-06	1.78E-06	1.78E-06	1.78E-06	1.78E-06	NAN
f3	3.90E-06	1.78E-06	1.78E-06	1.78E-06	1.78E-06	1.78E-06	1.78E-06	NAN
f4	1.78E-06	1.78E-06	1.78E-06	1.78E-06	1.78E-06	1.78E-06	1.78E-06	NAN
f5	NAN	1.78E-06	7.15E-06	1.78E-06	1.78E-06	1.78E-06	1.78E-06	1.78E-06
f6	NAN	1.78E-06	1.78E-06	1.78E-06	1.78E-06	1.78E-06	1.78E-06	1.78E-06
f7	9.41E-03	1.78E-06	1.78E-06	3.98E-06	1.97E-06	9.94E-04	1.78E-06	8.46E-04
f8	1.49E-07	1.77E-06	1.78E-06	1.74E-06	1.78E-06	NAN	1.78E-06	1.76E-06
f9	NAN	1.78E-06	1.78E-06	NAN	1.67E-06	NAN	1.78E-06	NAN
f10	NAN	1.78E-06	1.77E-06	5.53E-05	1.52E-06	NAN	1.78E-06	NAN
f11	NAN	1.78E-06	1.71E-06	NAN	NAN	NAN	1.78E-06	NAN
f12	4.47E-08	1.78E-06	1.78E-06	1.77E-06	1.78E-06	1.78E-06	1.78E-06	1.78E-06
f13	NAN	1.78E-06	1.72E-06	1.78E-06	1.78E-06	1.78E-06	1.78E-06	1.78E-06
f14	4.88E-04	1.78E-06	1.78E-06	3.13E-02	1.78E-06	NAN	1.78E-06	NAN
f15	3.07E-04	1.78E-06	1.78E-06	1.78E-06	1.77E-06	1.78E-06	1.78E-06	NAN
f16	1.78E-06	1.78E-06	1.78E-06	1.78E-06	1.78E-06	1.78E-06	1.78E-06	NAN
f17	NAN	1.78E-06	1.78E-06	1.00E-06	8.90E-07	1.78E-06	NAN	NAN
f18	4.47E-08	1.78E-06	1.74E-06	1.33E-04	1.76E-06	NAN	1.75E-06	1.76E-06
f19	3.91E-03	4.47E-08	4.47E-08	1.00E-06	4.47E-08	NAN	4.47E-08	NAN
**+**	**11**	**19**	**19**	**17**	**18**	**12**	**18**	**7**
**-**	**0**	**0**	**0**	**0**	**0**	**0**	**0**	**0**
**≈**	**8**	**0**	**0**	**2**	**1**	**7**	**1**	**12**

According to Table 4, CRLPO outperformed PO, PSO, DE, and GWO on 2, 3, 4, and 7 benchmark test functions, respectively. Further, CRLPO was similar to PO on 1, 5, 6, 9, 10, 11, 12, and 13 benchmark test functions. With respect to WOA, SCA, HHO, and RLGWO, CRLPO found better results than these four algorithms on 15, 19, 12, and 5 benchmark test functions, respectively. In addition, CRLPO was similar to HHO and RLGWO on 8 and 16 benchmark test functions, respectively. However, it was not surpassed by HHO, PO, or WOA in any case. Moreover, CRLPO was similar to WOA on 9 and 11 benchmark test functions. According to Table 4, CRLPO did the best on the Friedman’s test.

Table 5 shows the results of pair-wise comparisons of CRLPO and other algorithms demonstrated at a 0.05 significance level with 100 dimensions. As illustrated in Table 5, the larger number in “+” field and the lower number in “-” field show that CRLPO was statistically significant and relatively better than the other optimization techniques.

**Fig 15:**
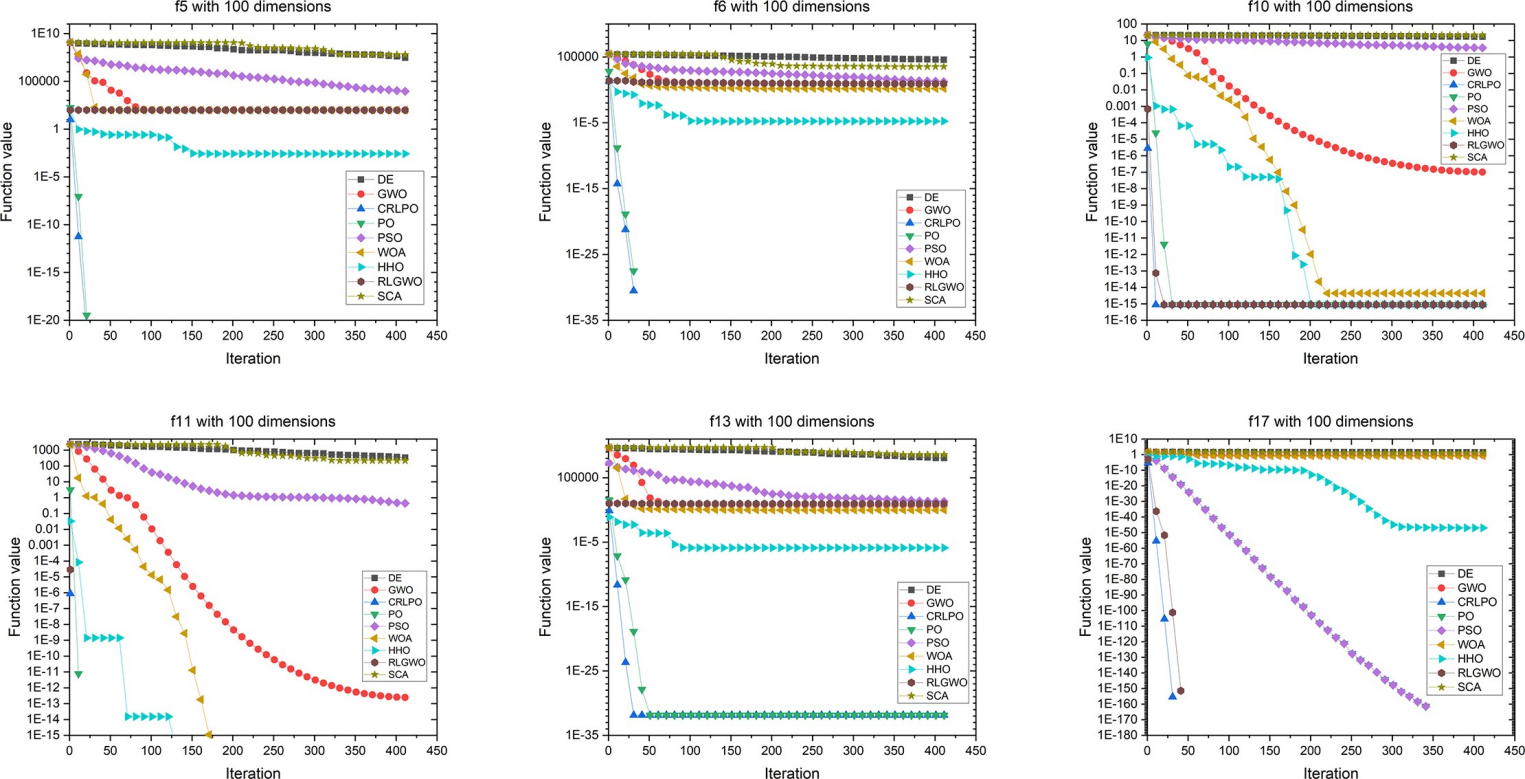
Convergence graph of CRLPO as well as eight other algorithms on six representative benchmark functions with 100 dimensions.

Fig 15 demonstrates an evolutionary process of the mean of the optimal value on six representative benchmark functions with 100 dimensions. As shown in Fig 15, CRLPO converged faster than the eight other optimization techniques in all six representative cases.

To further observe and study the scalability of CRLPO with large-scale problems, in experiment 4, CRLPO and the eight other algorithms were run independently thirty times on 19 benchmark functions (Table 1) with 1000 dimensions.

Table 6 summarizes the average (Ave) and standard (Std) deviation and results of the Friedman’s test with 1000 dimensions. Table 7 summarizes the p-values of the Wilconxon rank-sum test at a 0.05 significance level for CRLPO against the eight other algorithms with 1000 dimensions.

**Table 6:** Experimental results of 19 benchmark functions from Table 1 with 1000 dimensions.

F	Results	PO	PSO	DE	GWO	WOA	SCA	HHO	RLGWO	CRLPO
f1	Ave	0.00E+00	3.41E+04	2.05E+06	5.43E-01	6.37E-71	4.85E+05	6.01E-81	0.00E+00	0.00E+00
	Std	0.00E+00	1.68E+03	3.64E+04	1.55E-01	1.80E-70	1.30E+05	3.29E-80	0.00E+00	0.00E+00
f2	Ave	1.43E-175	1.39E+03	4.33E+03	1.11E+00	2.00E-43	INF	1.48E-44	0.00E+00	0.00E+00
	Std	0.00E+00	6.48E+01	6.94E+01	5.90E-01	7.14E-43	NAN	4.34E-44	0.00E+00	0.00E+00
f3	Ave	2.88E-128	1.80E+06	2.02E+07	1.24E+06	1.06E+08	2.51E+07	1.92E-38	0.00E+00	0.00E+00
	Std	1.58E-127	3.95E+05	1.44E+06	2.64E+05	3.57E+07	4.38E+06	9.10E-38	0.00E+00	0.00E+00
f4	Ave	3.76E-153	3.02E+01	9.92E+01	7.53E+01	8.51E+01	9.95E+01	2.41E-43	0.00E+00	0.00E+00
	Std	1.54E-152	9.06E-01	1.79E-01	3.45E+00	2.02E+01	1.19E-01	1.27E-42	0.00E+00	0.00E+00
f5	Ave	0.00E+00	2.38E+08	8.49E+09	1.08E+03	9.92E+02	4.64E+09	9.46E-02	9.97E+02	0.00E+00
	Std	0.00E+00	1.82E+07	2.11E+08	2.90E+01	5.54E-01	6.89E+08	1.52E-01	1.43E-01	0.00E+00
f6	Ave	0.00E+00	3.41E+04	2.04E+06	1.94E+02	3.25E+01	5.22E+05	1.52E-03	2.08E+02	0.00E+00
	Std	0.00E+00	1.27E+03	2.20E+04	2.61E+00	6.89E+00	1.57E+05	2.30E-03	2.46E+00	0.00E+00
f7	Ave	3.10E-04	2.37E+05	1.22E+05	1.20E-01	1.51E-03	7.06E+04	9.93E-05	8.55E-04	3.67E‐06
	Std	1.72E-04	7.38E+03	3.21E+03	2.50E-02	1.51E-03	1.39E+04	8.72E-05	1.05E-03	0.00E+00
f8	Ave	-4.15E+05	-4.08E+04	-7.97E+04	-9.49E+04	-3.82E+05	-2.29E+04	‐4.19E+05	-9.86E-04	‐4.19E+05
	Std	2.16E+04	8.75E+03	2.38E+03	2.14E+04	5.00E+04	1.65E+03	8.24E+00	4.93E+03	0.00E+00
f9	Ave	3.32E+01	1.39E+04	1.42E+04	2.51E+02	6.06E-14	1.85E+03	0.00E+00	0.00E+00	0.00E+00
	Std	1.82E+02	3.32E+02	1.18E+02	4.86E+01	3.32E-13	6.08E+02	0.00E+00	0.00E+00	0.00E+00
f10	Ave	8.88E‐16	1.52E+01	2.06E+01	2.88E-02	4.44E-15	1.98E+01	8.88E‐16	8.88E‐16	8.88E‐16
	Std	0.00E+00	2.90E-01	2.07E-02	3.84E-03	2.09E-15	3.00E+00	0.00E+00	0.00E+00	0.00E+00
f11	Ave	0.00E+00	1.43E+02	1.84E+04	4.56E-02	0.00E+00	4.67E+03	0.00E+00	0.00E+00	0.00E+00
	Std	0.00E+00	1.44E+01	2.54E+02	4.53E-02	0.00E+00	1.32E+03	0.00E+00	0.00E+00	0.00E+00
f12	Ave	4.71E‐34	6.04E+06	2.00E+10	1.20E+00	3.29E-02	1.42E+10	3.48E-07	8.13E-01	4.71E‐34
	Std	8.70E-50	1.35E+06	6.67E+09	2.88E-01	1.20E-02	2.23E+09	4.24E-07	1.90E-02	0.00E+00
f13	Ave	1.35E‐32	6.13E+07	3.74E+10	1.18E+02	1.74E+01	2.27E+10	1.78E-04	9.96E+01	1.35E‐32
	Std	5.57E-48	7.29E+06	9.98E+08	5.02E+00	6.08E+00	4.70E+09	2.35E-04	1.61E-01	0.00E+00
f14	Ave	9.80E-01	2.78E+02	3.14E+02	7.20E+01	9.00E‐01	3.57E+02	9.00E‐01	9.00E‐01	9.00E‐01
	Std	4.07E-02	5.81E+00	3.19E+00	1.30E+02	4.97E-16	3.92E+00	4.52E-16	4.52E-16	0.00E+00
f15	Ave	1.26E-176	1.53E+03	2.09E+03	1.20E+00	7.97E-45	2.68E+02	1.85E-42	0.00E+00	0.00E+00
	Std	0.00E+00	5.23E+01	2.24E+01	1.59E+00	2.72E-44	8.89E+01	8.76E-42	0.00E+00	0.00E+00
f16	Ave	1.24E-196	1.50E+256	INF	3.39E-03	2.47E-07	INF	8.76E-20	0.00E+00	0.00E+00
	Std	0.00E+00	INF	NAN	1.96E-02	6.67E-07	NAN	4.73E-19	0.00E+00	0.00E+00
f17	Ave	3.33E-03	2.90E+01	1.66E+02	1.97E+00	1.03E-01	6.74E+01	6.82E-45	0.00E+00	0.00E+00
	Std	1.82E-02	7.40E-01	9.59E-01	1.41E-01	6.68E-02	1.26E+01	1.50E-44	0.00E+00	0.00E+00
f18	Ave	‐3.92E+04	-1.07E+04	-1.27E+04	-1.34E+04	-3.82E+04	-8.42E+03	‐3.92E+04	-1.35E+04	‐3.92E+04
	Std	2.96E-11	6.49E+02	4.08E+02	7.60E+02	2.13E+03	4.58E+02	1.33E-01	8.70E+02	0.00E+00
f19	Ave	‐1.00E+00	2.65E-316	6.55E-279	3.40E-319	0.00E+00	1.03E-248	‐1.00E+00	‐1.00E+00	‐1.00E+00
	Std	0.00E+00	0.00E+00	0.00E+00	0.00E+00	0.00E+00	0.00E+00	0.00E+00	0.00E+00	0.00E+00
**Ave.R**		**2.32**	**7.16**	**8.37**	**5.89**	**4.53**	**7.95**	**2.63**	**2.68**	**1.00**
**Ova.R**		**2**	**7**	**9**	**6**	**5**	**8**	**3**	**4**	**1**

**Table 7:** p-values of the Wilconxon rank-sum test at 0.05 significance level for CRLPO against other eight algorithms on 19 benchmark functions from table 1 with 1000 dimensions.

Function	PO	DE	PSO	WOA	GWO	SCA	HHO	RLGWO
	P-value	P-value	P-value	P-value	P-value	P-value	P-value	P-value
f1	NAN	1.72E-06	1.77E-06	1.78E-06	1.78E-06	1.78E-06	1.78E-06	NAN
f2	1.78E-06	1.74E-06	1.71E-06	1.78E-06	1.78E-06	1.78E-06	1.78E-06	NAN
f3	1.78E-06	1.77E-06	1.77E-06	1.78E-06	1.77E-06	1.78E-06	1.78E-06	NAN
f4	1.78E-06	1.78E-06	1.78E-06	1.78E-06	1.78E-06	1.78E-06	1.78E-06	NAN
f5	NAN	1.78E-06	1.77E-06	1.78E-06	1.70E-06	3.18E-05	1.77E-06	1.78E-06
f6	NAN	1.63E-06	1.77E-06	1.78E-06	1.78E-06	1.78E-06	1.78E-06	1.78E-06
f7	2.60E-02	1.78E-06	1.78E-06	1.46E-05	1.78E-06	1.68E-02	1.78E-06	1.22E-03
f8	NAN	1.77E-06	1.78E-06	1.01E-02	1.78E-06	NAN	1.78E-06	1.77E-06
f9	NAN	1.03E-06	1.62E-06	NAN	1.78E-06	NAN	1.78E-06	NAN
f10	NAN	1.78E-06	1.78E-06	3.17E-06	1.78E-06	NAN	1.78E-06	NAN
f11	NAN	1.66E-06	1.78E-06	NAN	1.78E-06	NAN	1.78E-06	NAN
f12	NAN	1.75E-06	1.78E-06	1.78E-06	1.78E-06	1.78E-06	1.78E-06	1.78E-06
f13	NAN	1.77E-06	1.78E-06	1.78E-06	1.78E-06	1.78E-06	1.77E-06	1.78E-06
f14	1.00E-06	1.78E-06	1.78E-06	NAN	1.78E-06	NAN	1.78E-06	NAN
f15	4.88E-04	1.61E-06	1.74E-06	1.78E-06	1.78E-06	1.78E-06	1.78E-06	NAN
f16	1.78E-06	1.78E-06	1.78E-06	1.78E-06	1.78E-06	1.78E-06	1.78E-06	NAN
f17	NAN	1.78E-06	1.78E-06	7.20E-07	1.36E-06	1.78E-06	1.78E-06	NAN
f18	NAN	1.68E-06	1.74E-06	3.23E-06	1.76E-06	NAN	1.78E-06	1.72E-06
f19	NAN	4.47E-08	4.47E-08	4.47E-08	4.47E-08	NAN	4.47E-08	NAN
**+**	**7**	**19**	**19**	**16**	**19**	**12**	**19**	**7**
**-**	**0**	**0**	**0**	**0**	**0**	**0**	**0**	**0**
**≈**	**12**	**0**	**0**	**3**	**0**	**7**	**0**	**12**

According to Table 6, CRLPO outperformed PO, PSO, DE, and GWO on 2, 3, 4, and 7 benchmark test functions, respectively. In addition, CRLPO was similar to PO on 1, 5, 6, 10, 11, 12, 13, 18, and 19 benchmark test functions. With respect to WOA, SCA, HHO, and RLGWO, CRLPO found better results than these four algorithms on 8, 10, 12, and 18 benchmark test functions, respectively. Furthermore, CRLPO was similar to HHO and RLGWO on 9 and 17 benchmark test functions, respectively. However, it was not surpassed by HHO, PO, and WOA ion any case. Additionally, CRLPO was similar to HHO and 11 and 14 benchmark test functions. According to Table 6, CRLPO did the best on the Friedman’s test.

As shown in Table 7, the results of a pair-wise comparison of CRLPO and the other algorithms were demonstrated at a 0.05 significance level with 1000 dimensions. As illustrated in Table 7, the larger number in “+” field and the lower number in “-” field show that CRLPO was statistically significant and relatively better than the other optimization techniques.

**Fig 16:**
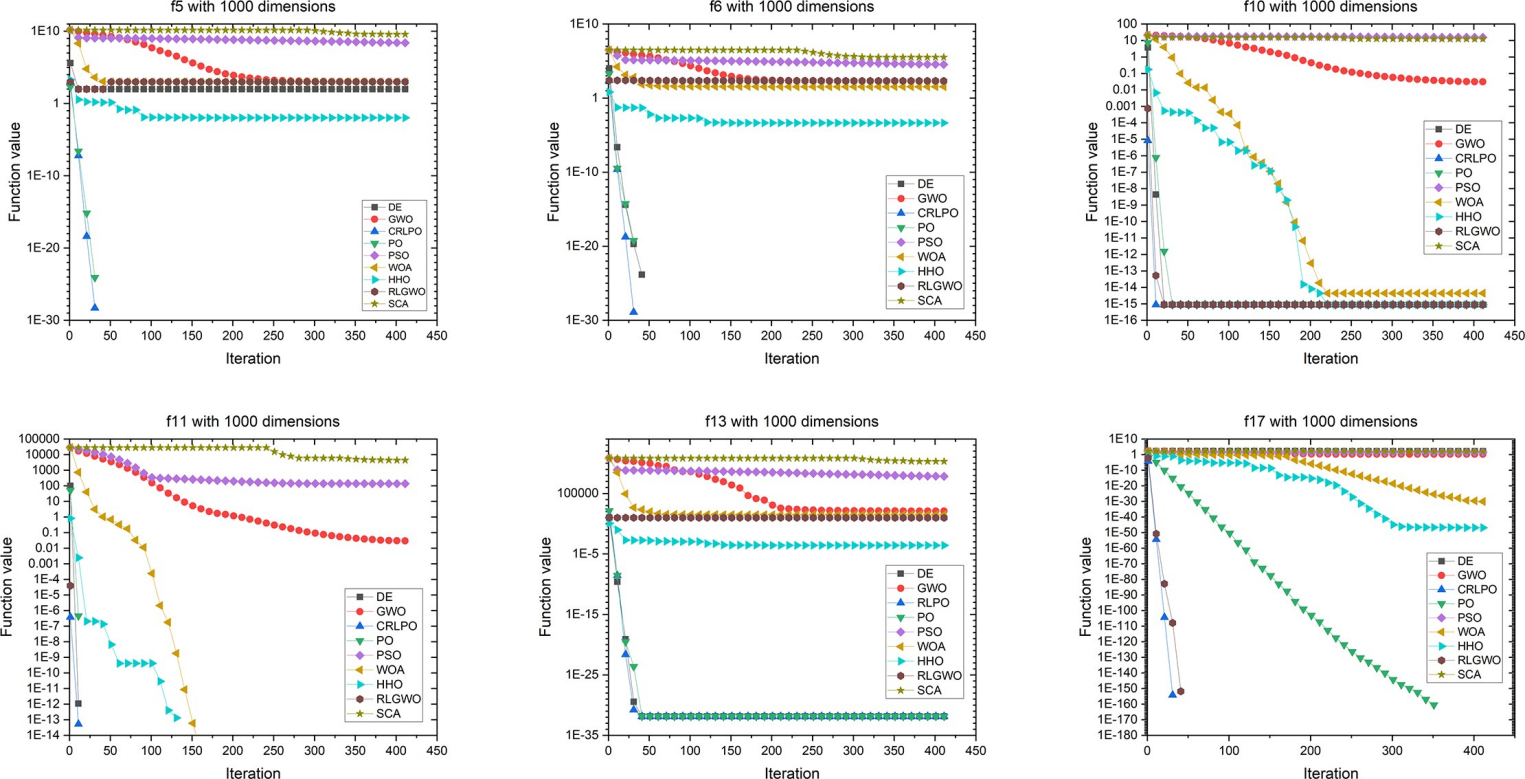
Convergence graph of CRLPO as well as eight other algorithms on six representative benchmark functions with 1000 dimensions.

Fig 16 demonstrates an evolutionary process of the mean of the optimal value on six representative benchmark functions with 1000 dimensions. As shown in Fig 16, CRLPO converged faster than the eight other optimization techniques in all six representative cases. To summarize, it was concluded that CRLPO had excellent scalability on test functions with 30D, 100D, and 1000D.

### Experiments on test functions from IEEE CEC 2014

To further evaluate the performance of CRLPO, other more complex 30 test functions from IEEE CEC 2014 were adopted. The IEEE CEC 2014 test suite can be separated into four categories. The first category (FC01- FC03) is unimodal functions, the second category (FC04- FC16) is multimodal functions, the third category (FC17- FC22) is hybrid functions, whereas the fourth category (FC23- FC30) is composition functions. The detailed problem definitions of 30 test functions from IEEE CEC 2014 were demonstrated in [65]. The search range of 30 test functions were defined as [–100, 100], and their dimensions were defined as 30.

To investigate the effectiveness of CRLPO, it was compared with nine state-of-the-art optimization techniques, including RW-GWO [66], CMA-ES [67], CLPSO [68], CoDE [69], MoABC [70], DGSTLBO [71], HCSA [72], LX-BBO [73], and RLGWO [58]. The parameter settings of the nine algorithms were the same as described in their original papers. To maintain the fairness of comparison, the same maximum number of function evaluations was set, which was 3.00E+10. CRLPO was run independently on 30 test functions.

The experimental results of the other nine optimization techniques were reviewed [58]. The error values (F(x)-F(x0)) were compared following 30 independent runs, where x is best position after an algorithm ends and x0 is the global optima. Table 8 shows the average of the error value (Ave), standard deviation (Std), and the results of the Friedman test. If an algorithm exhibited the best performance on a test function, the Ave results are displayed in bold face with a gray background.

**Table 8:** Comparison of CRLPO and nine selected algorithms on 30 test functions with 30 dimensions from IEEE CEC 2014.

F	Results	CMA-ES	CLPSO	CoDE	MoABC	DGSTLBO	HCSA	LX-BBO	RWGWO	RLGWO	CRLPO
F1	Ave	9.42E+04	1.48E+08	1.21E+07	2.81E+07	1.04E+07	3.50E+07	1.01E+07	8.02E+06	1.78E+07	2.69E+06
	Std	7.89E+04	2.57E+07	4.48E+06	1.01E+07	8.61E+06	2.49E+07	3.81E+06	3.31E+06	9.62E+06	1.37E+06
F2	Ave	2.55E+10	6.81E+09	1.89E+07	2.88E+04	4.59E+06	1.95E+07	5.34E+04	2.23E+05	1.14E+08	9.35E+03
	Std	3.85E+09	1.12E+09	9.45E+06	4.11E+04	1.11E+07	5.49E+07	2.14E+04	5.51E+05	3.86E+07	1.11E+04
F3	Ave	1.45E+04	9.86E+04	4.16E+03	1.06E+04	1.44E+01	3.10E+04	1.64E+04	3.16E+02	2.66E+04	6.29E+03
	Std	5.66E+03	1.68E+04	1.89E+03	3.66E+03	1.68E+01	1.36E+04	1.71E+04	4.34E+02	1.79E+03	3.56E+02
F4	Ave	2.52E+03	9.77E+02	1.44E+02	1.59E+02	1.46E+02	2.03E+02	9.99E+01	3.41E+01	1.69E+03	5.46E+01
	Std	5.36E+02	1.39E+02	1.55E+01	2.76E+01	3.78E+01	6.69E+01	2.85E+01	1.80E+01	2.38E+02	3.00E+01
F5	Ave	2.00E+01	2.11E+01	2.10E+01	2.04E+01	2.10E+01	2.00E+01	3.06E+00	2.05E+01	2.01E+01	2.02E+01
	Std	2.63E-05	4.87E-02	6.56E-02	3.53E.02	4.34E-02	2.28E-03	7.87E-01	7.46E-02	3.41E-02	1.54E-01
F6	Ave	4.09E+01	5.08E+01	5.57E+01	3.78E+01	1.67E+01	3.23E+01	1.69E+01	9.84E+00	3.64E+00	4.62E+00
	Std	2.13E+00	2.46E+00	2.67E+00	2.65E+00	3.45E+00	3.27E+00	3.12E+00	3.49E+00	9.55E-01	3.47E+00
F7	Ave	2.31E+02	6.32E+01	1.20E+00	5.72E-01	1.01E+00	1.79E+00	1.76E-01	2.53E-01	1.62E+02	1.30E‐03
	Std	2.83E+01	8.63E+00	7.20E-02	1.36E-01	1.50E+00	2.19E+00	8.56E-02	1.43E-01	2.65E+01	3.47E-03
F8	Ave	2.83E+02	2.92E+02	2.30E+02	1.26E+01	7.67E+01	1.71E+02	5.53E+01	4.38E+01	3.43E+01	2.69E+01
	Std	2.21E+01	1.87E+01	1.45E+01	1.74E+00	2.45E+01	3.46E+01	3.78E+02	8.48E+00	3.75E+01	7.32E+00
F9	Ave	3.28E+02	4.73E+02	3.80E+02	2.58E+02	9.84E+01	2.80E+02	7.66E+01	6.33E+01	1.21E+02	4.71E+01
	Std	3.47E+01	2.13E+01	1.89E+01	2.83E+01	3.08E+01	5.16E+01	1.61E+01	1.30E+01	0.00E+00	1.26E+01
F10	Ave	2.61E+02	7.62E+03	7.26E+03	2.29E+02	2.39E+03	2.66E+03	1.26E+04	9.61E+02	1.58E+03	1.12E+03
	Std	1.06E+02	5.19E+02	3.84E+02	1.07E+02	4.71E+02	5.34E+02	1.16E+04	2.72E+02	3.11E+02	6.88E+02
F11	Ave	1.69E+02	1.14E+04	1.218+04	5.74E+03	3.93E+03	4.13E+03	1.23E+04	2.68E+03	2.47E+03	2.63E+03
	Std	1.98E+02	5.09E+02	4.27E+02	3.27E+02	5.45E+02	5.35E+02	3.42E+02	3.68E+02	2.39E+02	8.23E+02
F12	Ave	3.03E-01	2.67E+00	2.47E+00	4.71E-01	2.75E+00	5.11E-01	1.11E‐02	5.45E-01	2.80E-01	4.57E-02
	Std	2.18E+00	3.28E-01	2.74E-01	5.73E-02	2.62E-01	2.56E-01	1.75E-18	1.66E-01	8.37E-02	4.81E-02
F13	Ave	5.51E+00	7.61E-01	6.53E-01	4.51E-01	4.71E-01	4.81E-01	6.55E-01	2.80E‐01	2.80E‐01	3.43E-01
	Std	3.07E-01	8.47E-02	6.56E-02	4.11E-02	1.13E-01	1.17E-01	1.56E-01	6.30E-02	1.30E-01	4.30E-02
F14	Ave	7.53E+01	1.60E+01	4.31E-01	2.98E-01	2.88E-01	3.08E-01	6.20E-01	4.23E-01	2 40E‐01	4.15E-01
	Std	8.08E+00	3.71E+00	8.50B-02	2.50E-02	4.92E-02	5.64E-02	2.96E-01	2.1SE-01	1.14E-01	1.95E-01
F15	Ave	1.02E+04	3.31E+03	3.78E+01	3.14E+01	3.75E+01	9.80E+01	1.55E+01	8.81E+00	3.68E+00	1.24E+01
	Std	3.24E+04	1.93E+03	2.26E+00	6.02E+00	2.19E+01	3.02E+01	5.50E+00	1.51E+00	2.06E+00	1.98E+00
F16	Ave	1.38E+01	2.24E+01	2.28E+01	1.97E+01	1.11E+01	1.27E+01	1.08E+01	1.03E+01	4.48E+00	1.21E+01
	Std	5.31E-01	2.33E-01	3.26E-01	4.02E-01	6.62E-01	5.01E-01	5.84E-01	6.11E-11	1.48E-01	2.09E-01
F17	Ave	5.49E+03	1.77E+07	1.81E+05	1.01E+07	1.67E+05	1.48E+06	1.46E+06	5.71E+05	1.50E+06	3.00E+05
	Std	3.62E+03	4.74E+06	1.24E+05	4.96E+06	2.13E+05	1.21E+06	9.34E+05	4.10E+05	2.49E+06	2.10E+05
F18	Ave	1.52E+09	2.51E+07	3.62E+03	9.92E+03	8.71E+02	7.67E+03	2.90E+03	6.52E+03	1.90E+06	2.28E+04
	Std	3.93E+08	8.22E+06	2.31E+03	9.94E+03	1.02E+03	6.70E+03	4.27E+03	4.62E+03	8.25E+05	6.87E+03
F19	Ave	2.98E+02	9.23E+01	3.62E+01	3.33E+01	2.71E+01	5.33E+01	5.19E+03	1.14E+01	6.12E+00	5.44E+00
	Std	4.25E+01	1.19E+01	1.08E+01	1.06E+01	2.86E+01	3.63E+01	5.67E+03	2.03E+00	2.03E+00	1.10E+00
F20	Ave	4.61E+03	5.17E+04	5.04E+02	3.96E+04	4.28E+02	3.93E+04	2.61E+04	6.27E+02	2.07E+04	6.31E+03
	Std	3.88E+03	1.06E+04	3.17E+02	1.29E+04	1.77E+02	2.20E+04	1.56E+04	1.12E+03	8.70E+03	2.38E+03
F21	Ave	6.86E+03	5.71E+06	2.12E+04	7.30E+06	2.20E+04	3.54E+05	1.11E+06	2.58E+05	1.44E+05	1.27E+05
	Std	2.76E+03	2.15E+06	1.61E+04	4.36E+06	2.22E+04	3.48E+05	7.95E+05	1.76E+05	6.25E+04	5.23E+04
F22	Ave	1.61E+03	1.36E+03	1.44E+03	1.14E+03	3.14E+02	9.47E+02	1.88E+03	2.08E+02	1.86E+02	2.17E+02
	Std	9.15E+01	1.71E+02	1.59E+02	1.89E+02	1.41E+02	3.31E+02	2.04E+02	1.29E+02	6.93E+01	1.72E+02
F23	Ave	5.79E+02	3.90E+02	3.55E+02	3.57E+02	3.15E+02	3.29E+02	4.11E+02	3.15E+02	2.00E+02	2.00E+02
	Std	4.94E+01	8.19E+00	1.77E-01	7.30E+00	4.43E-01	7.51E+00	6.43E+01	2.77E-01	0.00E+00	0.00E+00
F24	Ave	2.12E+02	3.39E+02	2.83E+02	2.71E+02	2.00E+02	2.78E+02	1.48E+04	2.00E+02	2.00E+02	2.00E+02
	Std	7.49E+00	6.72E+00	1.80E+00	1.78E+00	9.68E-04	3.11E+01	8.37E+03	3.04E-03	0.00E+00	0.00E+00
F25	Ave	2.12E+02	2.46E+02	2.18E+02	2.22E+02	2.02E+02	2.23E+02	5.29E+02	2.04E+02	2.00E+02	2.00E+02
	Std	2.97E+00	5.30E+00	1.94E+00	2.80E+00	3.62E+02	9.39E+00	4.37E+01	1.18E+00	0.00E+00	0.00E+00
F26	Ave	1.2SE+02	1.10E+02	1.04E+02	1.01E+02	1.10E+02	1.00E+02	2.13E+00	1.00E+02	1.00E+02	1.36E+02
	Std	5.51E+01	2.83E+01	1.82E+01	6.758–02	3.15E+01	1.63E-01	3.46E+00	7.36E-02	6.37E-02	4.79E+01
F27	Ave	1.07E+03	1.33E+03	1.28E+03	1.08E+03	7.94E+02	4.27E+02	1.96E+02	4.09E+02	2.00E+02	2.00E+02
	Std	2.30E+02	3.66E+02	1.47E+02	3.78E+02	2.15E+02	1.96E+01	1.04E+02	6.09E+00	0.00E+00	0.00E+00
F28	Ave	2.79E+03	3.02E+03	1.92E+03	2.15E+03	1.43E+03	3.49E+03	1.94E+03	4.34E+02	2.00E+02	2.00E+02
	Std	5.92E+02	4.20E+02	1.26E+02	3.42E+02	4.37E+02	5.48E+02	5.49E+02	8.45E+00	0.00E+00	0.00E+00
F29	Ave	3.52E+04	4.10E+05	2.00E+04	3.32E+03	3.08E+06	5.44E+05	1.98E+07	2.14E+02	2.00E+02	2.00E+02
	Std	5.34E+03	1.64E+05	7.15E+03	1.46E+03	4.99E+06	2.61E+06	3.96E+06	2.37E+00	0.00E+00	0.00E+00
F30	Ave	6.48E+05	6.00E+04	1.97E+04	1.61E+04	6.47E+03	2.49E+04	6.96E+06	6.69E+02	2.00E+02	2.00E+02
	Std	1.31E+05	1.52E+04	2.00E+03	4.10E+03	3.43E+03	2.26E+04	1.03E+07	2.14E+02	0.00E+00	0.00E+00
**Ave.R**		**6.8**	**8.63**	**6.3**	**5.73**	**4.85**	**6.6**	**5.9**	**3.3**	**3.7**	**2.86**
**Ova.R**		**9**	**10**	**7**	**5**	**4**	**8**	**6**	**2**	**3**	**1**

As illustrated in Table 8, CRLPO performed better than CMA-ES, CLPSO, CoDE, MoABC, and DGSTLBO on 2,7,9,19, and 23 benchmark functions, respectively. On the contrary, CMA-ES, CoDE, and DGSTLBO outperformed CRLPO on 1, 8, and 3 benchmark functions, respectively. Compared with MoABC, RW-GWO, and RLGWO, CRLPO exhibited improved and similar results on 1, 2, 7, 13, and 25 benchmark functions, respectively. In contrast, MoABC, RWGWO, and RLGWO beat CRLPO on 8, 4, and 6 benchmark functions, respectively. According to Table 8, CRLPO ranked first for the Friedman test.

Based on the analysis of the experimental results (Tables 2–8), it was concluded that the proposed CRLPO achieved competitive performance compared with other state-of-the-art algorithms.

### Effectiveness evaluation of two components of CRLPO

As described in section three, there were two components that comprised CRLPO (refraction learning, and adaptive parameter based on logistic model). To evaluate the effectiveness of these two components, additional experiments were required to be implemented. Firstly, we adopted RL only without adaptive parameters based on a logistic model in canonical PO. In this situation, parameter adjustment based on linear model in original PO was adopted, where such algorithm is defined as CRLPO-I. Secondly, we adopted an adaptive parameter based on a logistic model only without RL in canonical PO, where such an algorithm is defined as CRLPO-II. If both components are not adopted (parameter adjustment based on linear model), such algorithm is defined as RLPO-III. If both components and cubic interpolation are adopted, such algorithm is defined as CRLPO, as designed in section three.

Each algorithm was independently run on 19 test functions (Table 1) thirty times. The setting of the population size, as well as the maximum number of iterations were the same as those of section 4.3. As shown in Table 9, “+” represents the number of functions where an optimization technique significantly outperformed CRLPO, “-” indicates the number of functions where an optimization technique was statistically surpassed by CRLPO, and “≈” denotes the number of functions where CRLPO was similar to an algorithm. As illustrated in Table 9, the larger number in the “-” field and the lower number in “+” field reveal that CRLPO was statistically significant and relatively improved over the other algorithms.

**Table 9:** Experimental results of effectiveness evaluation of two components of CRLPO on 19 test functions from Table 1 with 100 dimensions.

F	CRLPO-I	CRLPO- II	CRLPO- III	CRLPO
	Ave±Std	Ave±Std	Ave±Std	Ave±Std
f1	0.00E+00±0.00E+00	0.00E+00±0.00E+00	0.00E+00±0.00E+00	0.00E+00±0.00E+00
f2	0.00E+00±0.00E+00	1.55 E-228±0.00E+00	4.26E-178±0.00E+00	0.00E+00±0.00E+00
f3	0.00E+00±0.00E+00	5.63 E-221±0.00E+00	1.52E-241±0.00E+00	0.00E+00±0.00E+00
f4	0.00E+00±0.00E+00	8.14 E-208±0.00E+00	4.59E-156±1.99E-155	0.00E+00±0.00E+00
f5	3.25 E+00±1.78 E+01	0.00E+00±0.00E+00	0.00E+00±0.00E+00	0.00E+00±0.00E+00
f6	0.00E+00±0.00E+00	0.00E+00±0.00E+00	0.00E+00±0.00E+00	0.00E+00±0.00E+00
f7	3.15 E- 04±2.02 E- 04	2.16 E- 04±1.60 E -04	2.77E-04±2.40E-04	2.97E+05±0.00E+00
f8	-4.18E- 04±7.40 E-12	-40713.9054±3745.349	-4.15E+04±2.16E+03	‐4.19E+04±0.00E+00
f9	0.00E+00±0.00E+00	0.00E+00±0.00E+00	0.00E+00±0.00E+00	0.00E+00±0.00E+00
f10	8.88E‐16±0.00E+00	8.88E‐16±0.00E+00	8.88E‐16±0.00E+00	8.88E‐16±0.00E+00
f11	0.00E+00±0.00E+00	0.00E+00±0.00E+00	0.00E+00±0.00E+00	0.00E+00±0.00E+00
f12	4.71E-33±1.39E-48	4.71E-33±7.24 E-49	4.71E‐33±0.00E+00	4.71E‐33±0.00E+00
f13	1.34 E -32±5.56E-48	1.34 E -32±2.88 E-48	1.35E‐32±0.00E+00	1.35E‐32±0.00E+00
f14	9.00E-01±4.51 E-16	9.46E-01±5.07 E-01	9.50E-01±5.09E-02	9.00E‐01±0.00E+00
f15	0.00E+00±0.00E+00	5.06 E-177±0.00E+00	5.55E-179±0.00E+00	0.00E+00±0.00E+00
f16	0.00E+00±0.00E+00	1.25 E-191±0.00E+00	6.79E-195±0.00E+00	0.00E+00±0.00E+00
f17	0.00E+00±0.00E+00	0.00E+00±0.00E+00	3.33E-03±1.82E-02	0.00E+00±0.00E+00
f18	**-**3.91 E+03**±**1.85E-12	**-**3.91 E+03±1.85 E-12	-3.87E+03±2.58E+02	‐3.92E+03±0.00E+00
f19	‐1.00E+00±0.00E+00	-8.66E-01± 3.45E-01	-7.00E-01±4.66E-01	‐1.00E+00±0.00E+00
**+/-/≈**	**0/7/12**	**0/12/7**	**0/11/8**	**/**

As shown in Table 9, compared with CRLPO, CRLPO-II showed a poorer performance on 12 benchmark functions and did not have a better performance on any benchmark function. The reason was that CRLPO-II uses only a nonlinear conversion parameter strategy. Furthermore, CRLPO-II can easily be trapped into a local optimum. However, the nonlinear conversion parameter strategy cannot assist it with jumping out of local optimum. Consequently, there were significant differences in performance between CRLPO-II and CRLPO.

Additionally, compared with CRLPO, CRLPO-I had a similar performance on 12 benchmark functions and did not have a better performance on any benchmark function. Such a phenomenon was attributed to that fact that RL was more effective for improving the exploration capacity during the search process. Consequently, there are no significant differences in performance between CRLPO-I and CRLPO.

Moreover, compared with CRLPO, CRLPO-III showed a poorer performance on 11 benchmark functions and did not have better performance on any benchmark function. CRLPO-III could also be easily trapped into a local optimum. However, the linear conversion parameter strategy could not help it to jump out of local optimum. Consequently, there were significant differences in performance between CRLPO-III and CRLPO.

To summarize, we concluded that the two mentioned components were mutually beneficial for enhancing the performance of PO. Further, the adaptive parameters based on a logistic model resulted in fast convergence, while a RL strategy enhanced the exploration capacity.

### Sensitivity analysis of the parameters of ξ and *p*

As described in the first portion of section three, RL is highly effective and was adopted to enhance global exploration. Nevertheless, the parameters in RL such as ξ and p have essential impacts on the performance of CRLPO. Consequently, to evaluate the impacts of ξ and p on the performance of CRLPO, additional experiments needed to be done. Firstly, let both ξ and p be larger than 1000. We found that, in such case, the performance of CRLPO was almost unchanged. Therefore, we selected the parameters of ξ and p in the range of from 1–1000. Secondly, to reduce the number of experiments, we selected a few typical values (e.g., 1, 10, 100, and 1000). Naturally, there were 16 combinations of ξ and p.

According to Eqs (34) and (35), we concluded that the combinations of both ξ = 1 p = 10 and ξ = 10 p = 1 had the same experimental results, and other combinations had the same effects. Therefore, as shown in Table 10, there were only seven representative combinations. The setting of population size, as well as the maximum number of iterations were the same as those of section 4.3. The dimensions were set to 30D, 100D, and 1000D, respectively (Tables 10–12).

**Table 10:** Experimental results of CRLPO for 19 test functions with typical combinations of ξ and p(Dim = 30).

F	Results	*ξ* = 1	*ξ* = 1	*ξ* = 1	*ξ* = 1	*ξ* = 10	*ξ* = 100	*ξ* = 1000
		*p* = 1	*p* = 10	*p* = 100	*p* = 1000	*p* = 1000	*p* = 1000	*p* = 1000
f1	Mean	0.00E+00	0.00E+00	0.00E+00	0.00E+00	0.00E+00	0.00E+00	0.00E+00
	St.dev	0.00E+00	0.00E+00	0.00E+00	0.00E+00	0.00E+00	0.00E+00	0.00E+00
f2	Mean	7.50E-232	0.00E+00	0.00E+00	0.00E+00	0.00E+00	0.00E+00	0.00E+00
	St.dev	0.00E+00	0.00E+00	0.00E+00	0.00E+00	0.00E+00	0.00E+00	0.00E+00
f3	Mean	0.00E+00	0.00E+00	0.00E+00	0.00E+00	0.00E+00	0.00E+00	0.00E+00
	St.dev	0.00E+00	0.00E+00	0.00E+00	0.00E+00	0.00E+00	0.00E+00	0.00E+00
f4	Mean	1.43E-208	0.00E+00	0.00E+00	0.00E+00	0.00E+00	0.00E+00	0.00E+00
	St.dev	0.00E+00	0.00E+00	0.00E+00	0.00E+00	0.00E+00	0.00E+00	0.00E+00
f5	Mean	0.00E+00	0.00E+00	0.00E+00	0.00E+00	0.00E+00	0.00E+00	0.00E+00
	St.dev	0.00E+00	0.00E+00	0.00E+00	0.00E+00	0.00E+00	0.00E+00	0.00E+00
f6	Mean	0.00E+00	0.00E+00	0.00E+00	0.00E+00	0.00E+00	0.00E+00	0.00E+00
	St.dev	0.00E+00	0.00E+00	0.00E+00	0.00E+00	0.00E+00	0.00E+00	0.00E+00
f7	Mean	3.25E-04	1.63E-04	8.37E-05	1.33E-04	1.51E-04	4.30E-04	5.63E-05
	St.dev	0.00E+00	0.00E+00	0.00E+00	0.00E+00	0.00E+00	0.00E+00	0.00E+00
f8	Mean	-1.26E+04	-1.26E+04	-1.26E+04	-1.26E+04	-1.26E+04	-1.26E+04	-1.26E+04
	St.dev	0.00E+00	0.00E+00	0.00E+00	0.00E+00	0.00E+00	0.00E+00	0.00E+00
f9	Mean	0.00E+00	0.00E+00	0.00E+00	0.00E+00	0.00E+00	0.00E+00	0.00E+00
	St.dev	0.00E+00	0.00E+00	0.00E+00	0.00E+00	0.00E+00	0.00E+00	0.00E+00
f10	Mean	8.88E-16	8.88E-16	8.88E-16	8.88E-16	8.88E-16	8.88E-16	8.88E-16
	St.dev	0.00E+00	0.00E+00	0.00E+00	0.00E+00	0.00E+00	0.00E+00	0.00E+00
f11	Mean	0.00E+00	0.00E+00	0.00E+00	0.00E+00	0.00E+00	0.00E+00	0.00E+00
	St.dev	0.00E+00	0.00E+00	0.00E+00	0.00E+00	0.00E+00	0.00E+00	0.00E+00
f12	Mean	1.57E-32	1.57E-32	1.57E-32	1.57E-32	1.57E-32	1.57E-32	1.57E-32
	St.dev	0.00E+00	0.00E+00	0.00E+00	0.00E+00	0.00E+00	0.00E+00	0.00E+00
f13	Mean	1.35E-32	1.35E-32	1.35E-32	1.35E-32	1.35E-32	1.35E-32	1.35E-32
	St.dev	0.00E+00	0.00E+00	0.00E+00	0.00E+00	0.00E+00	0.00E+00	0.00E+00
f14	Mean	1.00E+00	9.00E-01	9.00E-01	9.00E-01	9.00E-01	9.00E-01	9.00E-01
	St.dev	0.00E+00	0.00E+00	0.00E+00	0.00E+00	0.00E+00	0.00E+00	0.00E+00
f15	Mean	9.84E-230	0.00E+00	0.00E+00	0.00E+00	0.00E+00	0.00E+00	0.00E+00
	St.dev	0.00E+00	0.00E+00	0.00E+00	0.00E+00	0.00E+00	0.00E+00	0.00E+00
f16	Mean	7.26E-208	3.17E-290	0.00E+00	0.00E+00	0.00E+00	0.00E+00	0.00E+00
	St.dev	0.00E+00	0.00E+00	0.00E+00	0.00E+00	0.00E+00	0.00E+00	0.00E+00
f17	Mean	0.00E+00	0.00E+00	0.00E+00	0.00E+00	0.00E+00	0.00E+00	0.00E+00
	St.dev	0.00E+00	0.00E+00	0.00E+00	0.00E+00	0.00E+00	0.00E+00	0.00E+00
f18	Mean	-1.17E+03	-1.17E+03	-1.17E+03	-1.17E+03	-1.17E+03	-1.17E+03	-1.17E+03
	St.dev	0.00E+00	0.00E+00	0.00E+00	0.00E+00	0.00E+00	0.00E+00	0.00E+00
f19	Mean	-1.40E-43	-1.00E+00	-1.00E+00	-1.00E+00	-1.00E+00	-1.00E+00	-1.00E+00
	St.dev	0.00E+00	0.00E+00	0.00E+00	0.00E+00	0.00E+00	0.00E+00	0.00E+00

**Table 11:** Experimental results of CRLPO for 19 test functions with typical combinations of ξ and p (Dim = 100).

F	Results	*ξ* = 1	*ξ* = 1	*ξ* = 1	*ξ* = 1	*ξ* = 10	*ξ* = 100	*ξ* = 1000
		*p* = 1	*p* = 10	*p* = 100	*p* = 1000	*p* = 1000	*p* = 1000	*p* = 1000
f1	Mean	0.00E+00	0.00E+00	0.00E+00	0.00E+00	0.00E+00	0.00E+00	0.00E+00
	St.dev	0.00E+00	0.00E+00	0.00E+00	0.00E+00	0.00E+00	0.00E+00	0.00E+00
f2	Mean	1.78E-231	0.00E+00	0.00E+00	0.00E+00	0.00E+00	0.00E+00	0.00E+00
	St.dev	0.00E+00	0.00E+00	0.00E+00	0.00E+00	0.00E+00	0.00E+00	0.00E+00
f3	Mean	0.00E+00	0.00E+00	0.00E+00	0.00E+00	0.00E+00	0.00E+00	0.00E+00
	St.dev	0.00E+00	0.00E+00	0.00E+00	0.00E+00	0.00E+00	0.00E+00	0.00E+00
f4	Mean	2.64E-211	0.00E+00	0.00E+00	0.00E+00	0.00E+00	0.00E+00	0.00E+00
	St.dev	0.00E+00	0.00E+00	0.00E+00	0.00E+00	0.00E+00	0.00E+00	0.00E+00
f5	Mean	0.00E+00	0.00E+00	0.00E+00	0.00E+00	0.00E+00	0.00E+00	0.00E+00
	St.dev	0.00E+00	0.00E+00	0.00E+00	0.00E+00	0.00E+00	0.00E+00	0.00E+00
f6	Mean	0.00E+00	0.00E+00	0.00E+00	0.00E+00	0.00E+00	0.00E+00	0.00E+00
	St.dev	0.00E+00	0.00E+00	0.00E+00	0.00E+00	0.00E+00	0.00E+00	0.00E+00
f7	Mean	5.84E-04	9.39E-05	2.51E-04	1.87E-04	8.90E-05	2.97E-05	1.79E-04
	St.dev	0.00E+00	0.00E+00	0.00E+00	0.00E+00	0.00E+00	0.00E+00	0.00E+00
f8	Mean	-4.19E+04	-4.19E+04	-4.19E+04	-4.19E+04	-4.19E+04	-4.19E+04	-4.19E+04
	St.dev	0.00E+00	0.00E+00	0.00E+00	0.00E+00	0.00E+00	0.00E+00	0.00E+00
f9	Mean	0.00E+00	0.00E+00	0.00E+00	0.00E+00	0.00E+00	0.00E+00	0.00E+00
	St.dev	0.00E+00	0.00E+00	0.00E+00	0.00E+00	0.00E+00	0.00E+00	0.00E+00
f10	Mean	8.88E-16	8.88E-16	8.88E-16	8.88E-16	8.88E-16	8.88E-16	8.88E-16
	St.dev	0.00E+00	0.00E+00	0.00E+00	0.00E+00	0.00E+00	0.00E+00	0.00E+00
f11	Mean	0.00E+00	0.00E+00	0.00E+00	0.00E+00	0.00E+00	0.00E+00	0.00E+00
	St.dev	0.00E+00	0.00E+00	0.00E+00	0.00E+00	0.00E+00	0.00E+00	0.00E+00
f12	Mean	4.71E-33	4.71E-33	4.71E-33	4.71E-33	4.71E-33	4.71E-33	4.71E-33
	St.dev	0.00E+00	0.00E+00	0.00E+00	0.00E+00	0.00E+00	0.00E+00	0.00E+00
f13	Mean	1.35E-32	1.35E-32	1.35E-32	1.35E-32	1.35E-32	1.35E-32	1.35E-32
	St.dev	0.00E+00	0.00E+00	0.00E+00	0.00E+00	0.00E+00	0.00E+00	0.00E+00
f14	Mean	1.00E+00	9.00E-01	1.00E+00	9.00E-01	9.00E-01	9.00E-01	9.00E-01
	St.dev	0.00E+00	0.00E+00	0.00E+00	0.00E+00	0.00E+00	0.00E+00	0.00E+00
f15	Mean	2.53E-232	0.00E+00	0.00E+00	0.00E+00	0.00E+00	0.00E+00	0.00E+00
	St.dev	0.00E+00	0.00E+00	0.00E+00	0.00E+00	0.00E+00	0.00E+00	0.00E+00
f16	Mean	1.74E-209	1.2885e-311	0.00E+00	0.00E+00	0.00E+00	0.00E+00	0.00E+00
	St.dev	0.00E+00	0.00E+00	0.00E+00	0.00E+00	0.00E+00	0.00E+00	0.00E+00
f17	Mean	0.00E+00	0.00E+00	0.00E+00	0.00E+00	0.00E+00	0.00E+00	0.00E+00
	St.dev	0.00E+00	0.00E+00	0.00E+00	0.00E+00	0.00E+00	0.00E+00	0.00E+00
f18	Mean	-3.92E+03	-3.92E+03	-3.92E+03	-3.92E+03	-3.92E+03	-3.92E+03	-3.92E+03
	St.dev	0.00E+00	0.00E+00	0.00E+00	0.00E+00	0.00E+00	0.00E+00	0.00E+00
f19	Mean	3.08E-72	-1.00E+00	-1.00E+00	-1.00E+00	-1.00E+00	-1.00E+00	-1.00E+00
	St.dev	0.00E+00	0.00E+00	0.00E+00	0.00E+00	0.00E+00	0.00E+00	0.00E+00

**Table 12:** Experimental results of CRLPO for 19 test functions with typical combinations of ξ and p (Dim = 1000).

F	Results	*ξ* = 1	*ξ* = 1	*ξ* = 1	*ξ* = 1	*ξ* = 10	*ξ* = 100	*ξ* = 1000
		*p* = 1	*p* = 10	*p* = 100	*p* = 1000	*p* = 1000	*p* = 1000	*p* = 1000
f1	Mean	0.00E+00	0.00E+00	0.00E+00	0.00E+00	0.00E+00	0.00E+00	0.00E+00
	St.dev	0.00E+00	0.00E+00	0.00E+00	0.00E+00	0.00E+00	0.00E+00	0.00E+00
f2	Mean	6.45E-234	0.00E+00	0.00E+00	0.00E+00	0.00E+00	0.00E+00	0.00E+00
	St.dev	0.00E+00	0.00E+00	0.00E+00	0.00E+00	0.00E+00	0.00E+00	0.00E+00
f3	Mean	0.00E+00	0.00E+00	0.00E+00	0.00E+00	0.00E+00	0.00E+00	0.00E+00
	St.dev	0.00E+00	0.00E+00	0.00E+00	0.00E+00	0.00E+00	0.00E+00	0.00E+00
f4	Mean	5.89E-204	0.00E+00	0.00E+00	0.00E+00	0.00E+00	0.00E+00	0.00E+00
	St.dev	0.00E+00	0.00E+00	0.00E+00	0.00E+00	0.00E+00	0.00E+00	0.00E+00
f5	Mean	0.00E+00	0.00E+00	0.00E+00	0.00E+00	0.00E+00	0.00E+00	0.00E+00
	St.dev	0.00E+00	0.00E+00	0.00E+00	0.00E+00	0.00E+00	0.00E+00	0.00E+00
f6	Mean	0.00E+00	0.00E+00	0.00E+00	0.00E+00	0.00E+00	0.00E+00	0.00E+00
	St.dev	0.00E+00	0.00E+00	0.00E+00	0.00E+00	0.00E+00	0.00E+00	0.00E+00
f7	Mean	4.61E-06	3.18E-05	4.57E-05	2.75E-04	1.23E-04	3.68E-06	2.95E-04
	St.dev	0.00E+00	0.00E+00	0.00E+00	0.00E+00	0.00E+00	0.00E+00	0.00E+00
f8	Mean	-4.19E+05	-3.01E+05	-4.19E+05	-4.19E+05	-3.01E+05	-4.19E+05	-4.19E+05
	St.dev	0.00E+00	0.00E+00	0.00E+00	0.00E+00	0.00E+00	0.00E+00	0.00E+00
f9	Mean	0.00E+00	0.00E+00	0.00E+00	0.00E+00	0.00E+00	0.00E+00	0.00E+00
	St.dev	0.00E+00	0.00E+00	0.00E+00	0.00E+00	0.00E+00	0.00E+00	0.00E+00
f10	Mean	8.88E-16	8.88E-16	8.88E-16	8.88E-16	8.88E-16	8.88E-16	8.88E-16
	St.dev	0.00E+00	0.00E+00	0.00E+00	0.00E+00	0.00E+00	0.00E+00	0.00E+00
f11	Mean	0.00E+00	0.00E+00	0.00E+00	0.00E+00	0.00E+00	0.00E+00	0.00E+00
	St.dev	0.00E+00	0.00E+00	0.00E+00	0.00E+00	0.00E+00	0.00E+00	0.00E+00
f12	Mean	4.71E-34	4.71E-34	4.71E-34	4.71E-34	4.71E-34	4.71E-34	4.71E-34
	St.dev	0.00E+00	0.00E+00	0.00E+00	0.00E+00	0.00E+00	0.00E+00	0.00E+00
f13	Mean	1.35E-32	1.35E-32	1.35E-32	1.35E-32	1.35E-32	1.35E-32	1.35E-32
	St.dev	0.00E+00	0.00E+00	0.00E+00	0.00E+00	0.00E+00	0.00E+00	0.00E+00
f14	Mean	1.00E+00	9.00E-01	9.00E-01	9.00E-01	9.00E-01	9.00E-01	9.00E-01
	St.dev	0.00E+00	0.00E+00	0.00E+00	0.00E+00	0.00E+00	0.00E+00	0.00E+00
f15	Mean	0.00E+00	0.00E+00	0.00E+00	0.00E+00	0.00E+00	0.00E+00	0.00E+00
	St.dev	0.00E+00	0.00E+00	0.00E+00	0.00E+00	0.00E+00	0.00E+00	0.00E+00
f16	Mean	5.01E-207	6.42e-323	0.00E+00	0.00E+00	0.00E+00	0.00E+00	0.00E+00
	St.dev	0.00E+00	0.00E+00	0.00E+00	0.00E+00	0.00E+00	0.00E+00	0.00E+00
f17	Mean	0.00E+00	0.00E+00	0.00E+00	0.00E+00	0.00E+00	0.00E+00	0.00E+00
	St.dev	0.00E+00	0.00E+00	0.00E+00	0.00E+00	0.00E+00	0.00E+00	0.00E+00
f18	Mean	-3.92E+04	-3.92E+04	-3.92E+04	-3.92E+04	-3.92E+04	-3.92E+04	-3.92E+04
	St.dev	0.00E+00	0.00E+00	0.00E+00	0.00E+00	0.00E+00	0.00E+00	0.00E+00
f19	Mean	-1.00E+00	-1.00E+00	-1.00E+00	-1.00E+00	-1.00E+00	-1.00E+00	-1.00E+00
	St.dev	0.00E+00	0.00E+00	0.00E+00	0.00E+00	0.00E+00	0.00E+00	0.00E+00

As illustrated in Tables 10–12, compared with ξ = 100 and p = 1000, CRLPO with ξ = 1 and p = 1 achieved a poorer performance on most of benchmark functions. For functions 12, 13, and 18 similar results were obtained for CRLPO with all combinations of p and ξ. Consequently, observing the results of all combinations of p and ξ, it was concluded that the parameter setting of ξ = 100 and p = 1000 for CRLPO was appropriate.

### The effect of interpolation strategy on RLPO

The RLPO with Cubic interpolation, Quadratic interpolation, Advance quadratic interpolation, Lagrange interpolation and Newton interpolation are defined as CRLPO, QIRLPO, AQIRLPO, LIRLPO and NIRLPO, respectively. In order to further explore the effect of interpolation strategy on RLPO, 17 test functions from [51] were adopted. All of the PO variants were run thirty times on each benchmark function with 10 and 30 dimensions, respectively. The experimental results are demonstrated as Tables 13 and 14, which indicate that Cubic interpolation can more efficiently enhancing the performance of RLPO overall.

**Table 13:** Experimental results of RLPO with various interpolation strategies on 17 test functions from [51] (Dim = 10).

F	Result	RLPO	NIRLPO	LIRLPO	QIRLPO	AQIRLPO	CRLPO
F1	Ave	4.722E-131	8.842E-132	4.525E-129	3.787E-134	2.052E-134	1.030E‐134
	Std	2.585E-130	4.509E-131	2.476E-128	1.976E-133	7.919E-134	1.784E-134
F2	Ave	6.015E-04	7.130E-04	4.767E-04	6.039E-04	1.583E-66	2.816E‐74
	Std	4.520E-04	5.147E-04	4.132E-04	4.311E-04	8.645E-66	4.877E-74
F3	Ave	6.589E-206	8.442E-199	6.388E‐207	7.188E-203	1.578E-128	1.340E-142
	Std	0.000E+00	0.000E+00	0.000E+00	0.000E+00	7.456E-128	0.000E+00
F4	Ave	1.473E-67	1.960E-67	1.990E-66	1.421E-67	1.173E-61	8.035E‐74
	Std	5.489E-67	7.478E-67	7.417E-66	7.411E-67	5.834E-61	5.235E-68
F5	Ave	2.343E-64	1.626E-64	7.485E-63	1.084E-62	4.580E-04	0.000E+00
	Std	5.261E-64	4.460E-64	3.515E-62	4.157E-62	2.105E-03	0.000E+00
F6	Ave	0.000E+00	0.000E+00	0.000E+00	0.000E+00	0.000E+00	0.000E+00
	Std	0.000E+00	0.000E+00	0.000E+00	0.000E+00	0.000E+00	0.000E+00
F7	Ave	-3.500E+01	-3.500E+01	-3.500E+01	-3.500E+01	4.452E‐04	7.621E-04
	Std	0.000E+00	0.000E+00	0.000E+00	0.000E+00	3.497E-04	0.000E+00
F8	Ave	1.334E-125	2.582E‐130	4.542E-124	1.398E-128	4.185E+03	4.190E+03
	Std	7.304E-125	1.074E-129	2.417E-123	7.469E-128	2.528E+01	0.000E+00
F9	Ave	3.931E-66	1.917E-70	1.985E-67	5.938E-69	0.000E+00	0.000E+00
	Std	2.153E-65	7.884E-70	1.086E-66	1.660E-68	0.000E+00	0.000E+00
F10	Ave	0.000E+00	0.000E+00	0.000E+00	0.000E+00	0.000E+00	0.000E+00
	Std	0.000E+00	0.000E+00	0.000E+00	0.000E+00	0.000E+00	0.000E+00
F11	Ave	8.575E-04	1.225E-05	1.417E-05	7.327E-04	0.000E+00	0.000E+00
	Std	4.697E-03	6.707E-05	6.490E-05	3.071E-03	0.000E+00	0.000E+00
F12	Ave	3.872E-68	2.747E-69	6.358E-67	1.130E-68	1.163E-68	1.091E‐70
	Std	1.586E-67	1.102E-68	3.104E-66	3.901E-68	3.660E-68	8.452E-70
F13	Ave	8.663E-02	8.958E-02	8.185E-02	9.229E-02	8.205E-02	9.584E‐03
	Std	2.556E-02	2.901E-02	2.806E-02	2.803E-02	2.326E-02	8.846E-03
F14	Ave	2.904E-52	3.356E-55	3.409E-56	1.951E‐57	1.924E+00	9.980E-01
	Std	1.590E-51	1.261E-54	1.863E-55	6.877E-57	2.791E+00	0.000E+00
F15	Ave	4.550E-42	2.239E-48	4.570E-47	7.477E-48	2.233E-48	2.523E‐52
	Std	1.437E-41	5.293E-48	1.436E-46	2.133E-47	4.839E-48	0.000E+00
F16	Ave	-9.000E-01	-9.000E-01	‐1.000E+00	‐1.000E+00	‐1.000E+00	‐1.000E+00
	Std	3.162E-01	3.162E-01	0.000E+00	0.000E+00	0.000E+00	0.000E+00
F17	Ave	9.843E+02	1.098E+03	6.819E+02	2.765E+02	6.299E+02	3.877E+02
	Std	1.946E+03	1.736E+03	8.266E+02	3.782E+02	7.329E+02	1.243E+02
**Ave.R**		**3.5882**	**3.0000**	**3.2353**	**2.8235**	**3.1765**	**1.7647**
**Ova.R**		**6**	**3**	**4**	**2**	**5**	**1**

**Table 14:** Experimental results of RLPO with various interpolation strategies on 17 test functions from [51] (Dim = 30).

F	Result	RLPO	NIRLPO	LIRLPO	QIRLPO	AQIRLPO	CRLPO
F1	Ave	1.636E-132	7.809E-124	5.162E-127	3.362E-132	5.227E-132	7.827E‐146
	Std	4.964E-132	2.470E-123	1.632E-126	9.074E-132	1.591E-131	0.000E+00
F2	Ave	7.540E-04	4.975E-04	5.179E-04	8.141E-04	4.851E-04	9.400E‐77
	Std	6.858E-04	2.044E-04	6.437E-04	7.773E-04	1.990E-04	0.000E+00
F3	Ave	4.486E-207	5.99E‐215	2.087E-214	2.433E-208	1.026E-209	6.176E-143
	Std	0.000E+00	0.000E+00	0.000E+00	0.000E+00	0.000E+00	0.000E+00
F4	Ave	3.356E-69	3.007E-69	9.063E-67	6.231E-67	1.400E-65	3.370E‐72
	Std	8.983E-69	9.501E-69	2.847E-66	1.970E-66	4.421E-65	0.000E+00
F5	Ave	1.052E-61	2.948E-61	7.220E-60	7.789E-62	2.432E‐62	8.983E-04
	Std	3.204E-61	8.823E-61	2.107E-59	2.187E-61	5.361E-62	0.000E+00
F6	Ave	0.000E+00	0.000E+00	0.000E+00	0.000E+00	0.000E+00	0.000E+00
	Std	0.000E+00	0.000E+00	0.000E+00	0.000E+00	0.000E+00	0.000E+00
F7	Ave	-1.550E+02	-1.550E+02	-1.550E+02	-1.550E+02	-1.550E+02	4.140E‐04
	Std	0.000E+00	0.000E+00	0.000E+00	0.000E+00	0.000E+00	0.000E+00
F8	Ave	2.559E‐124	2.757E-108	3.833E-120	4.812E-110	2.644E-124	4.190E+03
	Std	8.087E-124	8.717E-108	1.211E-119	1.522E-109	6.135E-124	0.000E+00
F9	Ave	2.486E-66	6.960E-67	6.162E-70	2.220E-68	9.355E-68	0.000E+00
	Std	5.520E-66	1.488E-66	1.334E-69	5.913E-68	2.231E-67	0.000E+00
F10	Ave	0.000E+00	0.000E+00	0.000E+00	0.000E+00	0.000E+00	0.000E+00
	Std	0.000E+00	0.000E+00	0.000E+00	0.000E+00	0.000E+00	0.000E+00
F11	Ave	2.632E-03	7.307E-01	8.218E-21	1.290E+00	1.580E-01	0.000E+00
	Std	8.324E-03	2.311E+00	2.599E-20	4.064E+00	4.216E-01	0.000E+00
F12	Ave	3.840E-67	5.352E-67	7.510E-65	7.563E-67	7.624E-63	3.213E‐68
	Std	8.183E-67	1.234E-66	2.055E-64	2.347E-66	2.411E-62	8.451E-68
F13	Ave	1.854E-01	1.859E-01	1.873E-01	1.893E-01	1.780E‐01	1.978E-01
	Std	1.187E-02	2.328E-02	1.394E-02	1.219E-02	1.418E-02	3.714E-03
F14	Ave	6.332E-66	5.775E-63	3.493E-63	7.112E-65	5.914E‐66	2.521E+00
	Std	9.936E-66	1.410E-62	1.069E-62	1.281E-64	1.049E-65	0.000E+00
F15	Ave	3.414E-24	1.456E-21	9.524E-19	1.653E-24	2.313E-24	4.762E‐26
	Std	1.079E-23	4.148E-21	3.012E-18	5.225E-24	7.284E-24	8.248E-26
F16	Ave	-9.217E-01	-8.994E-01	-1.000E+00	-7.902E-01	-9.219E-01	‐6.192E‐01
	Std	2.477E-01	3.160E-01	6.952E-15	4.176E-01	2.471E-01	4.573E-01
F17	Ave	7.118E+02	1.416E+03	2.556E+03	2.273E+02	5.156E+02	4.346E+02
	Std	1.077E+03	2.076E+03	3.875E+03	3.175E+02	1.081E+03	5.671E+02
**Ave.R**		**2.8823**	**3.3529**	**3.8235**	**3.1176**	**2.8235**	**2.5294**
**Ova.R**		**3**	**5**	**6**	**4**	**2**	**1**

## Conclusion

A novel variant of PO (referred to as CRLPO) was proposed to enhance current metaheuristic methods. Inspired by the advantages of interpolation strategy and the phenomenon of refraction in nature, a sequence of novel PO variants was suggested and the best variant with interpolation strategy and RL strategy was proposed to form a hybrid with the original PO. We observed that CRLPO improved the diversity of the population and was beneficial for assisting PO with jumping out of local optimums. CRLPO was evaluated on 19 well-known benchmark functions with 30D, 100D, 1000D, and 30 benchmark functions from IEEE CEC 2014. The experimental results confirmed that CRLPO exhibited better, or at least competitive performance, when compared with selected state-of-the-art optimization techniques.
